# Herbal hepatotoxicity in traditional and modern medicine: actual key issues and new encouraging steps

**DOI:** 10.3389/fphar.2015.00072

**Published:** 2015-04-23

**Authors:** Rolf Teschke, Axel Eickhoff

**Affiliations:** Division of Gastroenterology and Hepatology, Department of Internal Medicine II, Klinikum Hanau, Academic Teaching Hospital of the Medical Faculty of the Goethe University Frankfurt MainFrankfurt, Germany

**Keywords:** herb induced liver injury, hepatotoxicity, herbal hepatotoxicity, herbs, evidence based trials, herbal traditional Chinese medicine, herbal modern medicine, herbal traditional medicine

## Abstract

Plants are natural producers of chemical substances, providing potential treatment of human ailments since ancient times. Some herbal chemicals in medicinal plants of traditional and modern medicine carry the risk of herb induced liver injury (HILI) with a severe or potentially lethal clinical course, and the requirement of a liver transplant. Discontinuation of herbal use is mandatory in time when HILI is first suspected as diagnosis. Although, herbal hepatotoxicity is of utmost clinical and regulatory importance, lack of a stringent causality assessment remains a major issue for patients with suspected HILI, while this problem is best overcome by the use of the hepatotoxicity specific CIOMS (Council for International Organizations of Medical Sciences) scale and the evaluation of unintentional reexposure test results. Sixty five different commonly used herbs, herbal drugs, and herbal supplements and 111 different herbs or herbal mixtures of the traditional Chinese medicine (TCM) are reported causative for liver disease, with levels of causality proof that appear rarely conclusive. Encouraging steps in the field of herbal hepatotoxicity focus on introducing analytical methods that identify cases of intrinsic hepatotoxicity caused by pyrrolizidine alkaloids, and on omics technologies, including genomics, proteomics, metabolomics, and assessing circulating micro-RNA in the serum of some patients with intrinsic hepatotoxicity. It remains to be established whether these new technologies can identify idiosyncratic HILI cases. To enhance its globalization, herbal medicine should universally be marketed as herbal drugs under strict regulatory surveillance in analogy to regulatory approved chemical drugs, proving a positive risk/benefit profile by enforcing evidence based clinical trials and excellent herbal drug quality.

## Introduction

Ancient Chinese and Egyptian papyruses describe medicinal use of plants for various ailments as early as 3.000 BC and thereby long before recorded history (Major, 1954; IARC Monographs, 2002). Starting at around that time, herbal traditional medicine originating from Mesopotamia, Egypt, and India influenced Byzantine, Greek, Latin, and Indian herbal medicine, thereby establishing principles of Ayurveda in India and developing traditional occidental herbal medicine, which subsequently became popular in numerous European and other Western countries (Major, 1954; IARC Monographs, 2002; Raghavendra et al., 2009; NIH, 2013). Indigenous cultures such as African, North American, Middle and South American, Australian, and South Pacific islandic also used herbs in their healing rituals (IARC Monographs, 2002). In other parts of the world, herbal traditional Chinese medicine (TCM) emerged (IARC Monographs, 2002; Raghavendra et al., 2009) and influenced the traditional Oriental herbal medicine in Japan (IARC Monographs, 2002), called Kampo medicine (Nishimura et al., 2009), and other Asian countries such as Korea with its herbal traditional Korean medicine (Park et al., 2012). With TCM originating in China and Ayurveda in India, two most ancient yet living traditions of herbal medicine presently remain and experience some extension over the globe (Patwardhan et al., 2005). Although, most other ancient herbal medicine cultures vanished or are restricted to local use without potential globalization, many countries use herbal medicines (WHO, 2002, 2013).

The worldwide use of medicinal herbs has increased over the past years (WHO, 2002, 2013; Ekor, 2014), but their regulatory surveillance differ among countries due to lack of harmonization (WHO, 2005; Ekor, 2014). The world market for herbal medicines based on traditional knowledge is estimated at US$60,000 million annually (WHO, 2002), according to a UN report dating back to 2000 (UN, 2000). Alone in the United States, the total estimated herb retail sales in all channels rose from $4230 million in 2000 to $6032 million in 2013, corresponding to 42.6% overall and to 3.3% on an annual basis according to the data of the American Botanical Council (Lindstrom et al., 2014). These figures compare to the increased use of complementary and alternative Medicine (CAM), since an estimated $27 billion was spent by consumers of CAM in the United States in 1997 (Eisenberg et al., 1998) and $33.9 billion in 2007 (Nahin et al., 2009), equaling a rise of 25.5%. These figures considered all CAM related expenditures spent out of pocket on visits to CAM practitioners and purchases of CAM products, classes, and materials in the United States in 2007, with $14.5 billion spent on the purchase of nonvitamin, nonmineral, and natural products (Nahin et al., 2009); the widespread use of herbal medicine exerts a high economic power in our society with special financial benefits for herb producers, providers, and healers. Considering this enormous economic impact and the resulting expenditures, the question is whether these high costs as burden for the consumers and the society are warranted. In addition, herbal medicine is increasingly exposed to major pressure due to concerns of efficacy, safety (NIH, 2014a), and adverse reactions (Podsadzki et al., 2013; Ekor, 2014) such as liver injury (NIH, 2014a).

In this review article, we critically analyze actual key issues of herbal hepatotoxicity by herbal products of traditional and modern medicine and discuss future developments. The expressions of herbal hepatotoxicity and herb induced liver injury (HILI) are used synonymously. Some similarities of HILI exist with DILI (drug induced liver injury) (Teschke et al., 2013f).

## Methods

### Data sources and searches

We used the PubMed to identify publications on herbal hepatotoxicity and HILI which each provided hits of around 279.000 and 1.840.000. Our search was then further qualified and extended using additional keywords denoting herbal modern medicine and herbal TCM, providing additional hits. The first 100 hits of publications in each category were considered.

### Study selection

The focus of our search was on publications in English language, but relevant reports of other languages also were considered. The retrieved publications included case reports, case series and review articles and were analyzed whether they were appropriate and relevant for the topic of this article. Publications also were manually searched for additional publications not yet identified.

### Data extraction and quality assessment

Prior to our analysis, the publications were assessed regarding their scientific and clinical quality. Publications of relevance and good quality were preferred and considered for evaluation.

## Key issues

Herbal products in traditional and modern medicine are commonly perceived by the general population as well tolerated and devoid of major adverse reactions. One of the most important goals in clinical practice is to offer patients an efficient therapy for their ailment(s), without harming their health. However, efficacy and safety by the use of herbs in traditional and modern medicine are features that may apply to some herbs and patients but certainly not to others. Similar shortcomings are known from conventional chemical drugs, which also are not effective in all patients. The risk of rare adverse reactions occurring in various organs including the liver relates to both, herbs (NIH, 2014a) and synthetic drugs (NIH, 2014b).

### Hepatotoxicity case reports

#### General aspects

Basic knowledge of hepatotoxicity by drugs and numerous other chemicals was summarized by Hyman Zimmerman in his pioneering book 25 years ago, briefly mentioning already some herbs as culprits and referencing a few case reports of herbal hepatotoxicity (Zimmerman, 1999). Since then, many more HILI cases emerged, which previously were reported (Pittler and Ernst, 2003b) and recently analyzed in publications on herbal TCM preparations (Teschke, 2014; Teschke et al., 2014c, 2015b), other commonly used herbal products (Teschke et al., 2013f), and herbal and dietary supplements (HDS) (Halegoua-De Marzio et al., 2013; Teschke et al., 2013d; Navarro et al., 2014; Robles-Diaz et al., 2015).

#### Epidemiology

Epidemiology data of hepatotoxicity cases in connection with herbal use are crucial to assess, both in traditional and modern medicine. Actually, the true prevalence of herbal hepatotoxicity is the total number of HILI cases in the population at a given time (Teschke et al., 2013f). It represents an estimate of how common herbal hepatotoxicity is within a population and at a fixed time. Conversely, the incidence of herbal hepatotoxicity is expressed as the total number of new HILI cases during a certain period of time, divided by the number of individuals in the population initially at risk. Therefore, incidence differs from prevalence measuring new HILI cases; for chronic liver injury, these values may change.

Incidence commonly provides information about the risk of acquiring HILI, whereas prevalence signifies how widespread HILI is. The true prevalence and incidence of HILI (Navarro, 2009) and HDS (Navarro et al., 2014; Robles-Diaz et al., 2015) is unknown. Global epidemiology considerations of prevalence and incidence refer to all herbs contained in herbal drugs and herbal supplements, whereas specific epidemiology is restricted to one single herb. Global epidemiology data therefore may be used for health economy assessment whereas specific epidemiology data pertain to herbal product safety. For this purpose, true global prevalence and incidence of HILI still has to be determined through cohort studies or case control-studies, a difficult approach. For an appropriate assessment of the risks from a specific herbal product, there is lack of quantitative data for consumption of herbal products, number of HILI patients, and the population at risk. In addition, herbal product authentication is missing in most cases of suspected HILI and impedes causality assessment for the incriminated herb (Teschke et al., 2013f). Case underreporting and overdiagnosing also prevent determination of the true incidence; future studies will have to address these issues in order to provide firm data of prevalence and incidence in HILI.

For DILI by synthetic drugs, respective data are available: the estimated annual incidence rate of DILI at a coordinating center in Spain was 34.2 ± 10.7 cases per 10^6^ inhabitants (Andrade et al., 2005), and in a French study it was 13.9 ± 9 per 10^6^ inhabitants per year (Sgro et al., 2002).

#### Compilation of hepatotoxicity cases

For herbal TCM with potential liver injury, we identified 44 different TCM herbs and 21 herbal TCM mixtures, published in case reports and case series as provided by appropriate references (Table 1). These referenced reports present clinical case details, summarized in part also earlier (Teschke, 2014). The 12 most common Chinese herbal medicines with hepatotoxicity detailed in a recent review (Ma et al., 2014) are also included in the present compilation (Table 1).

**Table 1:** **Compilation of reported cases with suspected hepatotoxicity by herbal traditional Chinese medicine (TCM)**.

**Name with ingredients**	**Cases (*n*)**	**References**
**Ai Ye** *Artemisia argyi*	na	Ma et al., 2014
**An Shu Ling** *Lycopodium serratum* or, rarely, *Corydalis* specie*s*, *Panax ginseng*, Pseudo ginseng, or two species of *Stephania*	1	Haller et al., 2002
**Bai Fang** *Angelica sinensis*, *Cyperus rotundus*, Ginseng, *Ligusticum wallichii*, *Paeonia alba*, *Rehmannia glutinosa*	1	Estes et al., 2003
**Bai Shi Wan** *Atractylis*, *Carthamus tinctorius*, *Dalbergia odorifera*, *Dioscorea bulbifera*, *Glycyrrhiza*, *Lithospermum erythrorhizon*, *Paeonia suffruticosa*, *Polygonum multiflorum*, *Psoralea corylifolia*, *Salvia miltiorrhiza*; Endoconcha sepiae, Ganoderma lucidum (mushroom)	1	Talari et al., 2010
**Bai Xian Pi** *Dictamnus dasycarpus*	1	Perharic-Walton and Murray, 1992
	1	Kane et al., 1995
	1	Vautier and Spiller, 1995
	2	Yuen et al., 2006
	4	Jang et al., 2008
	14	Kang et al., 2008
	2	Sohn et al., 2008
***Bi Ma Zi*** *Rhicinus communis*	na	Ma et al., 2014
***Ban Tu Wan*** *Angelica sinensis, Chaenomeles*, *Codonopsis pilosula*, *Notopterygium*, *Polygonum multiflorum*, *Rehmannia*, *Schisandra*	1	Cortez et al., 2012
**Bo He** *Mentha haplocalyx*		
**Bo Ye Qing Niu Dan** *Tinospora crispa*	2	Sangsuwan et al., 2004
**Bofu Tsu Sho San** *Angelica*, *Atractylis*, *Cnidium*, *Gardenia*, *Ephedra*, *Forsythia*, *Glycyrrhhiza*, *Gypsum fibrosum*, *Ledebouriella*, *Mentha*, *Paeonia*, *Platycodon*, *Rheum*, *Schizonepeta*, *Scutellaria*, *Zingiber;* Kadinum (talcum powder), sodium sulfuricum	1	Motoyama et al., 2008
**Boh Gol Zhee** *Psoralea corylifolia*	1	Hwang et al., 2001
	1	Nam et al., 2005
	3	Cheung et al., 2009
**Cang Er Zi** *Xanthium sibiricum*	na	Chau, 2008;
	na	Ma et al., 2014
**Chang Shan** *Dichora febrifuga Lour*	na	Ma et al., 2014
**Chai Hu** *Bupleurum falcatum*	28	Lee et al., 2011
**Chaso** *Camellia sinensis*, *Cassia tora* (syn. *Senna*), *Crataegus*, *Chrysanthenum morifolium Ramat.*, *Lotus*, *Lycium barbarum*; N-nitroso-fenfluramine	27	Adachi et al., 2003
**Chi R Yun** *Breynia officinalis*	2	Lin et al., 2002
	19	Lin et al., 2003
**Chinese herbal mixtures (various)** *Dictamnus dasycarpus*, *Gentiana scabra, Hedyotis diffusa*, *Paeonia suffructicosa*, *Paris polyphylla*, *Rehmannia glutinosa*, *Smilax glabra*, *Sophora subprostrata*;	1	Perharic-Walton and Murray, 1992
*Angelica sinensis*, *Bupleurum chinese*, *Dictamnus dasycarpus*, *Paeonia suffructiosa*, *Philodendron chinese*, *Saposhnikovia divaricata*, *Shisandra chinesis*, *Shizonepeta tenuifolia*, *Tribulus terrestris*;	2	Kane et al., 1995
*Cocculus trilobus*, *Dictamnus dasycarpus*, *Eurysolen gracilis*, *Glycyrrhiza*, *Lophatherum*, *Paeonia*, *Potentilla*, *Rehmannia glutinosa*;	1	Vautier and Spiller, 1995
*Alisma plantago aquatica*, *Artemisia capillaris*, *Bupleurum*, *Chrysanthemum morifolium*, *Circuma*, *Gardenia jasminoidis*, *Gentiana scabra*, *Glycyrrhiza*, *Magnolia*, *Paeonia*, *Plantago asiatica*, *Saussurea lappa*	1	Yoshida et al., 1996
**Chuan Lian Zi** *Melia toosendan*	1	Yuen et al., 2006
**Ci Wu Jia** *Acanthopanax senticosus*	2	Sohn et al., 2008
**Da Chai Hu Tang** *Bupleurum falcatum*, *Ginseng*, *Glycyrrhiza glabra*, *Pinellia*, *Scutellaria*, *Zingiber officinale*, *Zizyphus jujuba*	1	Kamiyama et al., 1997
**Da Huang** *Rheum palmatum*	1	Yuen et al., 2006
**Du Huo** *Angelica archangelica*	1	Björnsson et al., 2013
**Fu Fang Qing Dai Wan** Angelica dahurica, *Isatis indigotica* (Indigo naturalis), *Massa medicata fermentata* (yeast), *Salvia milthiorrhiza*, *Smilax glabra*	1	Verucchi et al., 2002
**Gan Cao** *Glycyrrhiza uralensis*, syn. Liquorice	1	Yuen et al., 2006
**Ge Gen** *Pueraria lobata*, syn. Arrowroot	2	Kim et al., 2009
**He Huan Pi** *Albizia julibrissin*	na	Ma et al., 2014
**Ho Shou Wu** *Polygonum multiflorum*, syn. He Shou Wu	1	Yuen et al., 2006
	na	Ma et al., 2014
	1	Bae et al., 2010
**Hu Bohe You** *Mentha pulegium*, syn. Pennyroyal oil	na	Chau, 2008
**Hu Zhang** *Polygonum cuspidatum*	na	Chau, 2008
**Huang Qin** *Scutellaria baicalensis*	19	Gono et al., 2010
	2	Linnebur et al., 2010
	1	Yang et al., 2012a;
	1	Dhanasekaran et al., 2013
**Huang Yao Zi** *Dioscorea bulbifera*	na	Chau, 2008;
	na	Ma et al., 2014
**Hwang Geun Cho** *Corydalis speciosa*	1	Kang et al., 2009
**Ji Gu Cao** *Abrus cantoniensis*	1	Yuen et al., 2006
**Ji Ji** *Chloranthus serratus*	na	Chau, 2008
**Ji Xue Cao** *Centella asiatica*, syn. Gotu Kola	3	Jorge and Jorge, 2005
**Jiguja** *Hovenia dulcis*	1	Sohn et al., 2008
	1	Kang et al., 2008
	1	Kim et al., 2012
**Jin Bu Huan** *Lycopodium serratum* or, rarely, *Corydalis species*,	7	Woolf et al., 1994
*Panax ginseng*, Pseudo ginseng, or two species of *Stephania*	3	Horowitz et al., 1996
	1	Picciotti et al., 1998
	1	Divinsky, 2002;
	1	Haller et al., 2002
**Jue Ming Zi** *Cassia obtusifolia*, syn. *Senna obtusifolia*	1	Yuen et al., 2006
**Kamishoyosan** *Angelica sinensis*, *Atractylodes racea*, *Bupleurum falcatum*, *Gardenia*, *Glycyrrhiza glabra*, *Mentha haplocalyx*, *Moutan*, *Paeonia alba*, *Sclerotium Poriae Cocos*, *Zingiber officinale*	1	Inoue et al., 2011
**Kudzu** *Pueraria thunbergiana*	6	Kang et al., 2008
**Ku Lian Zi** Melia azedarach	na	Ma et al., 2014
**Lei Gong Teng** *Tripterygium wilfordii* Hook	na	Chau, 2008
	na	Ma et al., 2014
**Long Dan Xie Gan Tang** *Acebia*, *Alisma*, *Angelica sinensis*, *Bupleurum*, *Gardenia*, *Gentiana*, *Glycyrrhiza*, *Plantago*, *Rehmannia*, *Scutellaria*	17	Lee et al., 2011
**Lu Cha** *Camellia sinensis*, syn. Chinese green tea	1	Garcia-Moran et al., 2004
	1	Peyrin-Biroulet et al., 2004
	1	Gloro et al., 2005
	1	Bonkovsky, 2006
	1	Jimenez-Saenz and Martinez-Sanchez, 2006
	1	Bonkovsky, 2006
	1	Molinari et al., 2006
	5	Björnsson and Olsson, 2007
	3	García-Cortés et al., 2008
	34	Sarma et al., 2008
	36	Mazzanti et al., 2009
	1	Rohde et al., 2011
	47	Navarro et al., 2013
**Ma Huang** *Ephedra sinica*	1	Nadir et al., 1996
	1	Borum, 2001
	3	Estes et al., 2003
	1	Skoulidis et al., 2005
	1	Reuben et al., 2010
**Mao Guo Tian Jie Cai** *Heliotropium lasiocarpum*	4	Culvenor et al., 1986
**Onshido** *Aloe*, *Camellia sinensis*, *Crataegus*, *Gynostemma pentaphyllum makino*, *Raphanus*; N-nitroso-fenfluramine	141	Adachi et al., 2003
**Qian Li Guang** *Senecio scandens*	na	Chau, 2008;
	na	Ma et al., 2014
**Ren Shen** *Panax ginseng*	6	Kang et al., 2008
**Sairei To** *Alisma*, *Atractylis*, *Bupleurum*, *Cinnamomum*, *Ginseng*, *Glycyrrhiza*,	1	Aiba et al., 2007
*Pinellia*, *Polyporus*, *Poria*, *Scutellaria*, *Zingiber*, *Zizyphus*	1	Tsuda et al., 2010
**Shang Lu** *Phytolacca acinosa*	na	Ma et al., 2014
**Shen Min** Black cohosh, Burdock, Cayenne pepper, *Ginkgo biloba*, Horse chestnut, *Piper nigrum, Polygonum multiflorum*, *uva ursi*; biotin, collagen (hydrolyzed), niacin, pantothenic acid, silica (from plant sources), soy isoflavones, vitamin A, vitamin B_6_	1	Cárdenas et al., 2006
**Shi Can** *Teucrium chamaedrys*, syn. Germander	na	Chau, 2008
**Shi Liu Pi** *Pericarpium granati*	na	Chau, 2008
**Shou Wu Pian** *Achyranthes bidentata*, *Cuscuta chinensis*, *Eclipta prostrata*,	1	But et al., 1996
*Ligustrum lucidum*, *Lonicera japonica*, *Morus alba*, *Polygonum multiflorum*,	1	Park et al., 2001
*Psoralea corylifolia*, *Rehmannia glutinosa*, *Rosa laevigata*, *Sesemum indicum*,	1	Battinelli et al., 2004
*Siegesbeckia orientalis*	1	Panis et al., 2005
	3	Sohn et al., 2008
	1	Laird et al., 2008
	1	Furukawa et al., 2010
	1	Valente et al., 2010
	25	Jung et al., 2011
	1	Banarova et al., 2012
**Tian Hua Fen** *Trichosanthes kirilowii*	na	Chau, 2008
**Tu San Qi** *Gynura segetum*	2	Dai et al., 2006
	1	Chen et al., 2007
	1	Li et al., 2010
	52	Lin et al., 2011;
	116	Gao et al., 2012
**White flood** Qian Ceng Ta (*Huperzia serrata*), Wu Zhu Yu (*Evodia rutaecarpa*); beet root, caffein, cocoa bean, vinpocetine (from *Vinca* plant); acesulfame potassium, calcium silicate, carnitine tartrate, Carno-Syn® beta-alanine, citrulline, cryptoxanthin, folic acid, gamma-aminobutyric acid (GABA), glucuronolactone, selenium, L-norvaline, L-tyrosine, lutein, malic acid, ornithine, potassium gluconate, sucralose, sugar cane, watermelon flavor, zeaxanthin	1	Cohen et al., 2012
**Wu Bei Zi** *Galla chinensis*	na	Chau, 2008
**Xi Shu** *Camptotheca acuminata*	na	Chau, 2008
**Xian Si Zi** *Abrus Precatorius*	na	Ma et al., 2014
**Xiao Chai Hu Tang** *Bupleurum falcatum, Ginseng*, *Glycyrrhiza glabra*,	4	Itoh et al., 1995
*Pinellia tuber, Scutellaria baicalensis, Zingiber officinale*, *Zizyphus jujuba*	19	Lee et al., 2011
	1	Hsu et al., 2006
**Yin Chen Hao** *Artemisia capillaris*	7	Kang et al., 2008
	1	Sohn et al., 2008
**Zexie** *Alisma orientalis*	1	Yuen et al., 2006
**Zhen Chu Cao** *Phyllanthus urinaria*	1	Yuen et al., 2006

Data represent an update of cases retrieved from a selective literature search for published cases of herbal TCM associated with suspected hepatotoxicity (Teschke, 2014; Teschke et al., 2014c, 2015b) and of a recent compilation most common Chinese herbal medicines with hepatotoxicity (Ma et al., 2014). In some cases, causality for individual herbs and herbal mixtures was established using the CIOMS (Council for International Organizations of Medical Sciences) scale or its modifications, and by positive reexposure test results. For other cases, information was fragmentary and did not necessarily allow a firm causal attribution.

Other herbs and herbal products unrelated to TCM showed reported potential hepatotoxicity for 111 items (Table 2), presented as an update of an earlier compilation (Teschke et al., 2012h). Most of the actual 111 items identified single herbs, rarely mixtures with HDS as examples with various ingredients (Table 2). Numerous other HDS with assumed potential hepatotoxicity are listed in compilations of other reports published just recently (Bunchorntavakul and Reddy, 2013; Navarro et al., 2014; Robles-Diaz et al., 2015) and hence were not included in the present compilation (Table 2).

**Table 2:** **Compitalion of commonly used herbs and herbal products with reported hepatotoxicity**.

**Search items**	**Botanical names, ingredients, references**
*Acacia catechu*	see Ayurvedic herb
Asterceae family	*Adenostyles alliariae* (Sperl et al., 1995)
Aloe	*Aloe perfoliata var. vera* (Rabe et al., 2005; Kanat et al., 2006; Bottenberg et al., 2007; Yang et al., 2010)
*Amorphophallus Konjac*	see Hydroxycut®
Arrowroot	*Maranta aruninacea* or *Tacca leontopetaloides* (Kim et al., 2009)
*Atractylis gummifera*	see Distaff thistle
Ayurvedic herbs	*Psoralea corylifolia, Acacia catechu, Eclipta alba* or *Bacopa monnieri, Vetivexia zizaniodis* (Teschke and Bahre, 2009)
Babchi	*Psoralea corylifolia*, see also Ayurvedic herbs (Nam et al., 2005)
*Bacopa monnieri*	see Ayurvedic herbs
Bajiaolian	*Dysosma pleianthum* (Kao et al., 1992)
*Boronia Sm.*	see Pro-Lean®
Buchu Tea	*Agathosma betulina*, *Agathosma crenulata* (Engels at al., 2013)
Bush tea	*Crotalaria species* (Smith and Culvenor, 1981)
*Callile pis laureola*	see Impila
*Camellia sinensis*	see green tea, Exolise®, and Hydroxycut® see X-elles®
Cascara sagrada	*Rhamnus purshianus* (Nadir et al., 2000)
*Cassia angustifolia*	see Senna
*Centella asiatica*	see Gotu Kola, see Pro-Lean®
*Chamaerops humilis*	see Saw Palmetto
Chaparral	*Larrea tridentata*, *Larrea divariatica*
syn. Creosot	(Katz and Saibil, 1990; Centers of Disease Control and Prevention, 1992; Smith and Desmond, 1993; Alderman et al., 1994; Batchelor et al., 1995; Gordon et al., 1995; Sheikh et al., 1997; Haller et al., 2002; Estes et al., 2003)
*Chelidonium majus*	see Greater Celandine, see Lycopodium similiaplex®
Chinese herbs	Unknown or up to 12 ingredients
*Chlorophora species*	see Kambala tea
*Chrysanthemum leucanthemum*	see Oxeye Daisy
*Citrus aurantium*	see X-elles®
*Citrus paradisum*	see X-elles®
*Cyrana scolymus*	see X-elles®
*Cola nitida*	see Pro-Lean®
Coltsfoot	*Tussilago farfara* (Roulet et al., 1988)
Comfrey	*Symphytum officinale*, *Symphytum asperum, Symphytum uplandicum* (Ridker et al., 1985; Weston et al., 1987; Bach et al., 1989; Ridker and McDermott, 1989; Miskelly and Goodyer, 1992)
*Compositae species*	see Indian herbs
Creosot	see Chaparral
*Crotalaria species*	see Bush tea, see Rattlebox
*Cyperus*	see Pro-Lean®
Distaff thistle	*Atractylis gummifera* (Georgia, 1988)
*Eclipta alba*	see Ayurvedic herbs
*Emblica officinalis*	see Isabgol
*Ephedra species*	*Ephedra californica*, *Ephedra sinica* (Estes et al., 2003)
Exolise®	*Garcinia cambogia, Gymnema sylvestre*, White kidney bean, *Camellia sinensis*, L-Carnitine fumarate, Calcium, Magnesium chelate, Chromium chelate, Conjugated linoleic acid, Chitosan (McDonnell et al., 2009)
*Fallopia multiflora*	see Pro-Lean®
*Foeniculum amare*	see Herbalife®
*Fucus vesiculosus*	see Pro-Lean®
*Garcinia cambogia*	see Exilis®, see Herbalife®, see Hydroxycut®
Germander	*Teucrium chamaedrys*, *Teucrium polium* (Larrey et al., 1992; Mostefa-Kara et al., 1992; Dao et al., 1993; Mattéi et al., 1995; Laliberté and Villeneuve, 1996; Starakis et al., 2006)
*Ginkgo biloba*	see Pro-Lean®
*Ginseng*	see Bai Fang, see Dai Saiko To, see Pro-Lean®,
	see Xiao Chai Hu Tan
*Glycyrrhiza glabra*	see Dai Saiko To, see Xiao Chai Hu Tang
Gotu Kola	*Centella asiatica* (Jorge and Jorge, 2005)
Greater Celandine	*Chelidonium majus*, see also Lycopodium similiaplex® (Strahl et al., 1998; Greving et al., 1998; Benninger et al., 1999; Crijns et al., 2002; Hardeman et al., 2003; Stickel et al., 2003; BfArM, 2005; Rifai et al., 2006; Conti et al., 2008; Moro et al., 2009; Tarantino et al., 2009; EMA, 2010; Teschke et al., 2011a, 2012a, 2013b)
Green tea	*Camellia sinensis*, see also Lu Cha (Table 1) (Duenas Sadornil et al., 2004; Garcia-Moran et al., 2004; Abu el Wafa et al., 2005; Gloro et al., 2005; Bonkovsky, 2006; Javaid and Bonkovsky, 2006; Jimenez-Saenz and Martinez-Sanchez, 2006; Molinari et al., 2006; Martinez-Sierra et al., 2006; Björnsson and Olsson, 2007; Federico et al., 2007; Liss and Lewis, 2009; Sarma et al., 2008; Mazzanti et al., 2009; Verheist et al., 2009; Rohde et al., 2011; Teschke et al., 2011e, 2014e; Teschke and Schulze, 2012)
Groundsel	*Senecio longilobus, Senecio species*
syn. Senecio	Stillman et al., 1977; Fox et al., 1978
Guaraná	*Paullinia cupana* (Dara et al., 2008)
*Gymnema sylvestre*	see Exilis®, see Hydroxycut®
Hawthorn	see *Crataegus*
*Hedeoma pulegoides*	see Pennyroyal
Heliotropium	*Heliotropium eichwaldii*, *Heliotropium species* (Mohabbat et al., 1976; Datta et al., 1978; Tandon et al., 2008; Kakar et al., 2010)
Herbalife®	*Solidaginis gigantea, Ilex paraguariensis, Petroselinum crispum, Garcinia cambogia, Spiraea, Matricaria chamomilla, Liquiritia, Foeniculum amare, Humulus lupulus*, Chromium, and various other ingredients (Hoffmann et al., 2005; Duque et al., 2007; Elinav et al., 2007; Schoepfer et al., 2007; Chao et al., 2008; Manso et al., 2008; Stickel et al., 2009; Chen et al., 2010; Jóhannsson et al., 2010; Appelhans et al., 2011; Manso et al., 2011; Appelhans et al., 2012; Manso, 2012; Halegoua-De Marzio et al., 2013; Teschke et al., 2013b)
Horse chestnut	see Venencapsan®, see Venoplant®
Hydroxycut®	*Camellia sinensis*, *Gymnema sylvestre, Amorphophallus Konjac, Paullinia cupana*, *Garcinia cambogia*, Caffeine, α-Lipoic acid, L-Carnitine, Calcium, Potassium, Chromium (Stevens et al., 2005; Jones and Andrews, 2007; Dara et al., 2008; Shim and Saab, 2009; Chen et al., 2010; Fong et al., 2010)
*Humulus lupulus*	see Herbalife®
*Ilex paraguariensis*	see Herbalife®, see Maté
Impila	*Callilepis laureola* (Wainwright et al., 1977; Wainwright and Schonland, 1977; Popat et al., 2001)
Indian herbs	*Compositae species* (Kumana et al., 1983)
Iroko	see Kambala tea
Isabgol	*Plantago ovata, Emblica officinalis* (Fraquelli et al., 2000)
Kambala tea	*Chlorophora excelsa, Chlorophora regia*
syn. Iroko	(Gunawan and Kaplowitz, 2004)
Kava	*Piper methysticum* (Strahl et al., 1998; Escher et al., 2001; BfArM, 2002; Bujanda et al., 2002; Denham et al., 2002; Weise et al., 2002; Estes et al., 2003; Gow et al., 2003; Humberston et al., 2003; Russmann et al., 2003; Schulze et al., 2003; Teschke et al., 2003, 2008a,b, 2011b, 2012c; Schmidt et al., 2005; WHO, 2007a; Christl et al., 2009; Teschke and Wolff, 2009, 2011; Teschke, 2010a,c; Teschke and Schulze, 2010; Teschke and Lebot, 2011; Schmidt, 2014)
*Larrea divariatica*	see Chaparral
*Larrea tridentata*	see Chaparral
*Leucanthemum vulgare*	see Oxeye Daisy
*Liquiritia*	see Herbalife®
*Lycopodium serratum* foot clubmass	see Lycopodium similiaplex®, see Wolf's
Lycopodium similiaplex®	*Lycopodium serratum, Chelidonium majus* (Conti et al., 2008)
*Maranta aruninacea*	see Arrowroot
Maté	*Ilex paraguariensis* (McGee et al., 1976)
*Matricaria chamomilla*	see Herbalife®
*Mentha pulegium*	see Pennyroyal
Mistletoe	*Viscum album* (Harvey and Colin-Jones, 1981; Hyde, 1981; Colin-Jones and Harvey, 1982; Farnsworth and Loub, 1982; Stirpe, 1983)
*Monascus purpureus*	see Red Yeast Rice
*Morinda citrifolium*	see Noni
*Nerium oleander*	see Oleander
Noni	*Morinda citrifolium* (Millonig et al., 2005; Stadlbauer et al., 2005, 2008; Yüce et al., 2006; López-Cepero Andrada et al., 2007; Yu et al., 2011; Mrzljak et al., 2013)
Oleander	*Nerium oleander* (Altan et al., 2009)
Oxeye Daisy	*Leucanthemum vulgare, Chrysanthemum leucanthemum* (Mokhobo, 1976)
*Paullinia cupana*	see Guaraná, see Hydroxycut®, see Pro-Lean®
Pennyroyal	*Mentha pulegium*, *Hedeoma pulegoides* (Vallance, 1955; Sullivan et al., 1979; Anderson et al., 1996; Bakerink et al., 1996)
*Petroselinum crispum*	see Herbalife®
*Petroselinum sativum*	see X-elles®
*Piper methysticum*	see Kava
*Phaseolus vulgaris*	see Exilis ®
*Plantago ovata*	see Isabgol
Pro-Lean®	Ma Huang, *Paullinia cupana, Cola nitida*, *Centella asiatica, Salix alba*,*Ginkgo biloba, Fucus vesiculosus*, *Boronia Sm.*, *Ginseng*, *Fallopia multiflora*, *Cyperus*, Bee pollen, Caffeine, L-Tyrosine, Chromium, Vanadium, Magnesiumsalicylat, Folsäure, Vitamin B_12_, and various other ingredients (Joshi et al., 2007)
*Psoralea corylifolia*	see Ayurvedic herbs
Pyrrolizidine alkaloids	see Bush tea, see Comfrey, see Groundsel, see *Heliotropium species*,
	see Indian herbs, see Maté, see Rattlebox
Rattlebox	*Crotalaria species*
syn. *Crotalaria*	(Tandon et al., 1976a,b)
Red Yeast Rice	*Monascus purpureus* (Roselle et al., 2008)
*Rhamnus purshianus*	see Cascara sagrada
Rooibos Tea	*Aspalathus linearis* (Engels et al., 2013)
*Salix alba*	see Pro-Lean®
Sassafras	*Sassafras albidum* (Larrey, 1997; Zimmerman, 1999)
Saw Palmetto	*Serenoa serpens, Chamaerops humilis* (Lapi et al., 2010)
Scullcap	*Scutellaria lateriflora, Scutellaria species* (MacGregor et al., 1989; Caldwell et al., 1994; Hullar et al., 1999; Estes et al., 2003; Yang et al., 2012a)
*Scutellaria species*	see Scullcap
*Senecio*	see Groundsel
Senna	*Cassia angustifolia* (Beuers et al., 1991; Seybold et al., 2004; Vanderperren et al., 2005)
*Serenoa serpens*	see Saw Palmetto
*Solidaginis gigantea*	see Herbalife®
*Spiraea*	see Herbalife®
*Symphytum*	see Comfrey
*Tacca leontopetaloides*	see Arrowroot
*Teucrium*	see Germander
*Tussilago farfara*	see Coltsfoot
Valerian	*Valeriana officinalis* (MacGregor et al., 1989; Mennecier et al., 1999)
	
*Valeriana officinalis*	see Valerian
Venencapsan®	*Aesculus hippocastanum*, *Chelidonium majus*, *Melilotus officinalis*, Milfoil, *Silybum* Adans., *Taraxacum officinale* (De Smet et al., 1996)
Venoplant®	*Aesculus hippocastanum* (Takegoshi et al., 1986)
*Vetivexia zizaniodis*	see Ayurvedic herbs
*Viscum album*	see Mistletoe
Wolf's foot clubmass	*Lycopodium serratum* (Woolf et al., 1994; Horowitz et al., 1996; Conti et al., 2008)
X-elles®	*Petroselinum sativum, Citrus aurantium, Citrus paradisum, Cyrana scolymus, Camellia sinensis* (Mathieu et al., 2005)

Data are retrieved from a selective literature search for selective reports of herbs and herbal products with hepatotoxicity and actualized from a previous report (Teschke et al., 2012h). In numerous cases, causality was proposed, but not necessarily established and open for discussion.

In the past, some review articles focused exclusively on HILI by TCM herbs and herbal preparations (Ma et al., 2014; Teschke, 2014; Teschke et al., 2014c, 2015b) as a primarily neglected topic, which was otherwise considered as part of overall assessments on herbal hepatotoxicity in few publications (Zimmerman, 1999; Abdualmjid and Sergi, 2013; Bunchorntavakul and Reddy, 2013), including an official and well updated NIH statement (NIH, 2014a).

#### Symptomatology

Clinical symptoms of herbal hepatotoxicity in traditional and modern medicine are variable and described in published case reports, case series, and regulatory presented spontaneous reports as referenced (Tables 1, 2). Symptoms are mostly unspecific and sometimes difficult to direct to the liver, which delays early recognition of the unfolding liver injury (Teschke et al., 2013f, 2014c; Ma et al., 2014). Clinical signs may emerge alone or in combination with other features, while jaundice is the symptom initially best recognized by the patient, facilitating the search for advice by the primary care physician. In detail, patients with herbal TCM hepatotoxicity experience fatigue (67.3%), jaundice (60.3%), anorexia (58.0%), nausea (35.9%), and fever (19.3%), but signs such as rash, pruritus, and pale stools have also been reported (Ma et al., 2014). In another study of 16 cases of Greater Celandine (GC) with established HILI, symptoms were present in 15 cases (Teschke et al., 2012a). Single or multiple symptoms were anorexia (*n* = 3), fatigue (*n* = 5), nausea (*n* = 6), vomiting (*n* = 2), dyspepsia (*n* = 1), bloating (*n* = 1), abdominal discomfort (*n* = 1), right upper quadrant pains (*n* = 1), epigastric pains (*n* = 1), unspecified abdominal pains (*n* = 1), fever (*n* = 1), dark urine (*n* = 3), pale stool (*n* = 1), pruritus (*n* = 3), and jaundice (*n* = 15) (Teschke et al., 2012a). For reasons of transparency, narrative case details and clinical data of patients with assumed HILI should be provided in tabular form, as done previously (Teschke et al., 2008a, 2011a, 2012a,b,d,e; Teschke, 2010a) and shown for GC hepatotoxicity as example (Table 3). Detailed information also allows characterization of HILI by a single herb such as GC (Table 4).

**Table 3:** **Compilation of narrative case details and clinical data of patients with HILI by Greater Celandine (GC) and established causality**.

**Patient**	**Identification**	**Specific information for each individual patient**
01	(Strahl et al., 1998), 42 years Female	GC extract of known brand name and manufacturer (3 capsules/day containing each 200 mg of probably dried herb for 9 months). Bloating as indication for treatment. Latency period of 2 months for first symptoms of itching and jaundice, at rechallenge 1 month. ALT 755 U/L, AST 350 U/L, ALP 221 U/L. Upon cessation of GC treatment, rapid decrease but not normalization of ALT values reported. Readministration of GC with positive result. Exclusion of virus hepatitis A-C and infections of other hepatotropic viruses reported, with lack of any details regarding hepatitis A, B, and C, CMV, EBV, HSV, or VZV. Exclusion of biliary obstruction by sonography. Exclusion of autoimmune hepatitis reported with lack of specified parameters. Normal values of iron and copper parameters. Liver histology: Acute hepatitis with confluent liver cell necroses and little inflammation.
		**Final diagnosis: highly probable GC hepatotoxicity**.
02	(Benninger et al., 1999), their case five 37 years Female	GC extract as drug of known brand name and manufacturer (unknown dose/day for 3 months). Atopic eczema as indication for GC treatment. Various herbal and homeopathic drugs as CD. Latency period of 3 months until symptoms of nausea and jaundice. ALT 813 U/L, AST 898 U/L, ALP 249 U/L. ALT course described. Positive reexposure test for GC. Five months after GC discontinuation, normalization of liver parameters reported. Exclusion of infections by HAV, HBV, HBC, HEV, CMV, and EBV. SMA 1:40, exclusion of AIH. Ultrasound examination with normal bile ducts. Liver histology not done.
		**Final diagnosis: highly probable GC hepatotoxicity**.
03	(Benninger et al., 1999), their case six 65 years Female	GC extract of unknown brand name and manufacturer (unknown dose/day for 3 months). Dyspepsia as indication for GC treatment. Latency period and symptoms not recorded. No CD. ALT 152 U/L, AST 89 U/L, ALP 451 U/L. After GC discontinuation, ALT course not sufficiently documented. Normalization of liver values 3 months after GC withdrawal. Exclusion of common causes for hepatitis reported, but lack of information regarding specific parameters. Lack of ultrasound data. Liver histology: Moderate drug induced hepatitis with low grade single cell necrosis.
		**Final diagnosis: highly probable GC hepatotoxicity**.
04	(Crijns et al., 2002), 42 years Female	Herbal mixture of GC and curcuma root (*Curcuma longa rhizoma*) of known brand name and manufacturer (unknown dose/day for 2 months). Not further described skin complaints as indication for treatment. Before admission, paracetamol (500 mg) tablet on one day. Latency period: 5 weeks until jaundice. Fever 40.5°C for 2 weeks, starting 2 weeks after initiation of GC treatment. ALT 1490 U/L, AST 838 U/L, ALP 265 U/L. Following cessation of the herbal mixture, ALT course described with normalization of liver values after 2 months. Exclusion of acute hepatitis A-C and infections by CMV, and EBV, but HSV and VZV not assessed. Normal titres of ANA, but no data of AMA, SMA, and LKM. Sonography with normal biliary tract. Liver histology: Severe acute hepatitis of viral or toxic drug cause.
		**Final diagnoses: probable GC hepatotoxicity and possible curcuma hepatotoxicity**.
05	(Stickel et al., 2003), their case two 69 years Male	GC extract as drug of known brand name and manufacturer (80 capsules within 5–6 weeks). Postprandial abdominal discomfort as indication for GC treatment. Latency period of 5–6 weeks until symptoms of weakness, abdominal pain in the right upper quadrant, nausea, jaundice, and dark brown urine. Medical history included cholecystectomy 4 years ago. Lack of regular comedication. Alcohol consumption below 20 g/day. ALT 881 U/L, AST 466 U/L, ALP 312 U/L. ALT course not recorded. Exclusion of acute viral hepatitis including HAV, HBV, HCV, CMV, EBV. Autoimmune parameters not assessed. By abdominal ultrasound and magnetic resonance tomography common and intrahepatic bile ducts inapparent. Liver histology: Cholestatic hepatitis compatible with drug toxicity.
		**Final diagnosis: probable GC hepatotoxicity**.
06	(Rifai et al., 2006), 58 years Male	GC extract as drug of known brand name and manufacturer (unknown amounts of tablets/day for 3 weeks). Biliary spasms as indication for GC treatment. Latency period: of 3 weeks until fatigue, dark urine, itching, jaundice, and pale stool. No CD. ALT 903 U/L, AST 380 U/L, ALP 516 U/L. After GC withdrawal well documented ALT course with ALT normalization within 4 weeks. Well documented exclusion of hepatitis A–C, and E, and of infections by CMV, EBV, HSV, and VZV reported. Well documented exclusion of infectious, autoimmune, metabolic, and genetic causes of acute hepatitis. Sonography with slightly thickening of the gall bladder and otherwise normal biliary tract. Liver histology: Lobular hepatitis with severe cholestasis and moderate inflammation that included also the bile ducts.
		**Final diagnosis: probable GC hepatotoxicity, but also possible causality for biliary disease**.
07	(Conti et al., 2008), 46 years Female	GC extract as solution of known brand name and manufacturer, containing also other herbs as are *Lycopodium serrata, Carduus marianus, Hamamelis, Ruta, Sepia, Pulsatilla, Collinsonia, and Hydrastis* (50 drops/day for 8 weeks). Insomnia and for sedation as indication for treatment. Latency period of 8 weeks until symptoms of nausea, anorexia, asthenia, and abdominal discomfort. Herbal mixture with various herbs and the potentially hepatotoxic *Lycopodium serrata* as CD. ALT 2,364 U/L, AST 737 U/L, ALP 255 U/L. Rapid decrease of ALT in the further course following treatment cessation with normalization after 2 months. Exclusion of HAV, HBV, HCV, CMV, EBV, and HSV. Specified serological tests for autoimmune diseases negative. Sonography without reported biliary tract abnormalities. Liver histology: Moderate mixed inflammatory infiltrate with eosinophils.
		**Final diagnoses: probable GC hepatotoxicity, probable *Lycopodium serratum* hepatotoxicity**.
08	(Moro et al., 2009), 65 years Male	GC extract as herbal tea derived from GC leaves (1 cup/day for 1 month). Pyrosis as indication for GC treatment. Lansoprazole 15 mg/day for 2 years as current CD. Latency period of 1 month. Asthenia, dyspepsia, dark urine, and jaundice as symptoms. ALT 4765 U/L, AST 3235 U/L, ALP not reported. ALT course not reported, but normalization of all liver parameters within 2 months. Three months before symptom onset, treatment with clarithromycin and amoxicillin for 1 week. All antibodies for not further specified hepatic viruses resulted negative except for anti-HCV that was found positive despite negative HCV-PCR. Autoimmune parameters not reported. Hepatomegaly by ultrasound examination. Liver histology: Moderate drug induced hepatitis.
		**Final diagnosis: probable GC hepatotoxicity**.
09	BfArM, 2005, 95003848 32 years Male	GC extract as drug of known brand name and manufacturer (2 capsules/day for not clearly defined duration). Upper abdominal pains as indication for treatment. Latency period not report ed, jaundice as symptom. ALT 2196 U/L, AST 714 U/L, ALP 256 U/L. Upon cessation of GC treatment, decrease but not normalization of ALT and AST values, with lack of reported ALP value. Readministration of GC with pruritus and not further specified increases of liver values and lack of complete resolution upon dechallenge. Overall course of ALT not sufficiently documented, neither at first and second dechallenge, nor in the interval and after the second challenge. Undulating ALT values of unknown clinical significance. Exclusion of virus hepatitis reported, with lack of any details regarding hepatitis A, B, and C, CMV, EBV, HSV, or VZV. Exclusion of biliary obstruction by sonography and ERCP. Exclusion of autoimmune hepatitis with lack of reported parameters. Normal values of ceruloplasmin, α-1 Antitrypsin, and electrophoresis. Liver histology: Unspecific hepatitis with liver cell necroses. Poorly documented case including questionable rechallenge and lack of ALT normalization.
		**Final diagnosis: probable GC hepatotoxicity**.
10	BfArM, 2005, 96026841 55 years Female	GC extract as drug of known brand name and manufacturer (3 capsules/day for 6 weeks). Upper abdominal pains as indication for treatment. Latency period of 6 weeks with jaundice as symptom. Diltiazem 90 for several years and doxycycline for 10 days (start prior to jaundice) for treatment of erythema migrans as CD. ALT 2,016 U/L, AST 620 U/L, ALP 398 U/L. After cessation of GC treatment, normalization of ALT not reported and with 201 U/L on day 19 still increased. Overall ALT course poorly documented. Exclusion of hepatitis A, B, and C reported without details of assessed parameters. Lack of exclusion of virus infections by CMV, EBV, HSV, and VZV. Negative results for AMA, SMA, LKM, and actin. Exclusion of biliary obstruction by sonography and ERCP. Liver histology: compatible with drug induced liver injury
		**Final diagnosis: highly probable GC hepatotoxicity**.
11	BfArM, 2005, 98000501 65 years Male	GC extract as drug of known brand name and manufacturer (2–3 capsules/day for 42 days). To increase bile flow after cholecystectomy 20 years ago as indication for treatment. Latency period of 42 days with itching and jaundice as symptoms. ALT 461 U/L, AST 355 U/L, ALP 260 U/L, normalization not reported. After GC discontinuation, on day 12 ALT 235 U/L. Exclusion of hepatitis A–C and of infections by CMV, EBV, HSV, and VZV. AMA negative, exclusion of autoimmune hepatitis reported but individual parameters not described. Upon sonography and ERCP normal bile ducts after cholecystectomy.
		**Final diagnosis: highly probable GC hepatotoxicity**.
12	BfArM, 2005, 98001447 49 years Female	GC extract as drug of known brand name and manufacturer (3 tablets/day for 4 weeks). Upper abdominal pains as indication for treatment. Latency period of 3.5 weeks with reduced appetite, bloating, epigastric pain, nausea, vomiting, and jaundice as symptoms. ALT 2928 U/L, AST 1116 U/L, ALP 408 U/L. After GC discontinuation, on day 7 ALT was 1356 U/L, and on day 20 it was 426 U/L. Normalization of ALT has not been reported. Exclusion of hepatitis A-C, E and F, and of infections by CMV, EBV, and HSV, but not of VZV. Normal values of ANA, AMA, and SMA. Upon ultrasound and ERC, normal bile ducts and cholecystolithiasis or cholesterol polyps of the gallbladder, by ultrasound questionable cholecystitis. Liver histology: Severe portal hepatitis with beginning fibrosis.
		**Final diagnosis: probable GC hepatotoxicity**.
13	BfArM, 2005, 98001607 59 years Female	GC extract as drug of known brand name and manufacturer (3 tablets/day for 7 weeks). Vomiting, upper abdominal pains and gastro-esophageal reflux as indications for treatment. Latency period 20 days with tiredness, exhaustion, nausea, vomiting, and jaundice as symptoms. Asthma, treated with various sprays, and latent hyperthyroidism without treatment as comorbidities. Maximum values reported for ALT 960 U/L, AST 421 U/L, and ALP 425 U/L, with decrease but not normalization following GC discontinuation, but actual results have not been reported. Through histology, ERCP, and serology (HAV, HBV, HCV) other hepatobiliary diseases excluded, but details not reported. Missing exclusion of infections by CMV, EBV, HSV, and VZV. Poorly documented case.
		**Final diagnosis: probable GC hepatotoxicity**.
14	BfArM, 2005, 98008527 60 years Female	GC extract as drug of known name and manufacturer (3 capsules/day for several weeks). General discomfort as indication for treatment. Latency period of several weeks with abdominal pains, nausea, and jaundice as symptoms. Crataegus extract as CD. ALT 420 U/L, AST 451 U/L, ALP 288 U/L. At discharge after 4 weeks, ALT with 26 U/L still slightly elevated. Exclusion of acute hepatitis A–C and infections by CMV, and EBV, but HSV and VZV not assessed. Normal titres of ANA, AMA, SMA, and LKM. Sonography and ERCP with normal biliary tract. Liver histology: AIH or dug induced liver injury.
		**Final diagnosis: probable GC hepatotoxicity**.
15	(BfArM, 2005), 00000278 65 years Male	GC extract as drug of known brand name and manufacturer (3 capsules/day for 4 weeks). Bloating as indication for treatment. Latency period of 3.5 weeks with jaundice as symptom. Diclofenac (intermittent), sitosterols, butizide, raubasine, rescinnamine, and reserpine as CD. ALT 950 U/L, AST 570 U/L, normal ALP. Under treatment with cortisone and at discharge, ALT 193 U/L, but normalization of ALT with and without cortisone not reported. Exclusion of hepatitis A–C and of infections by CMV, EBV, and HSV reported. Normal titres of ANA, AMA, and SMA. Sonography and ERCP with normal biliary tract. Liver histology: Hepatitis with cholestasis.
		**Final diagnosis: probable GC hepatotoxicity**.
16	(BfArM, 2005), 02001171 66 years Female	GC extract as drug of known brand name and manufacturer (0–2 capsules/day for 4.5 months). Dyspepsia as indication for treatment. Latency period of 4.5 months with reduced appetite and jaundice as symptoms. ALT 760 U/L, AST 408 U/L, ALP 337 U/L. On day 14 after GC cessation, ALT 379 U/L, and on day 24 ALT 207 U/L. Normalization of ALT not reported. Exclusion of hepatitis A–C reported, but details not presented. No exclusion of infections by CMV, EBV, HSV, and VZV. Autoimmune parameters not done. Sonography, MRCP and MRT with normal biliary tract. Insufficiency of the mitral valve.
		**Final diagnosis: probable GC hepatotoxicity**.

Narrative compilation with details of relevant clinical data of 16 patients with liver injury by the use of the herb Greater Celandine (GC), data are derived from a review article (Teschke et al., 2012a) and based on previous reports (Teschke et al., 2011a, 2012e). In these studies, highly probable and probable causality levels for GC were established in all patients presented as final diagnoses, using the updated CIOMS scale for the individual causality assessment. Half of the patients (cases 01–08) were derived from published case reports, the other half (cases 09–16) from spontaneous reports of the German regulatory agency (BfArM, 2005). Outcome was favorable in all cases. Abbreviations: AIH, autoimmune hepatitis; ALP, alkaline phosphatase; ALT, alanine aminotransferase; AMA, antimitochondrial antibodies; ANA, antinuclear antibodies; AST, aspartate aminotransferase; BfArM, Bundesinstitut für Arzneimittel und Medizinprodukte, Bonn; CD, comedicated drug(s); CIOMS, Council for International Organizations of Medical Sciences; CMV, cytomegalovirus; EBV, Epstein Barr virus; ERCP, endoscopic retrograde cholangiopancreaticography; GC, Greater Celandine; HAV, hepatitis A virus; HBV, hepatitis B virus; HCV, hepatitis C virus; HSV, herpes simplex virus; LKM, liver kidney microsomal antibodies; MRCP, magnetic resonance cholangiopancreaticography; MRT, magnetic resonance tomography; PCR, polymerase chain reaction; SMA, smooth muscle antibodies; VZV, varicella zoster virus.

**Table 4:** **Preferred documentation as example: clinical characteristics of GC hepatotoxicity**.

**Characteristics of HILI by GC**
Characterization of GC hepatotoxicity as a specific disease entity was feasible and based on high causality levels for GC in 16 patients with liver disease.
Causality for GC was graded highly probable and probable in 4 and 12 patients, respectively.
Among these 16 patients, there was an additional causality for comedicated curcuma graded as possible, for comedicated Lycopodium serratum graded as probable, and for biliary disease graded as possible.
The existence of GC hepatotoxicity has been verified by a positive reexposure test in two patients
Ages of the 16 patients ranged from 32 to 69 years with an average of 54.7 years, and the ratio of females: males was 10: 6.
Comedication with synthetic or herbal drugs and dietary supplements including herbal ones and herbal mixtures was used in the majority of assessable cases.
On average, the patients used 10 mg chelidonine daily with lack of daily overdose in any of the cases.
Treatment duration was 3 weeks to 9 months with an average of 2.4 months.
Latency period until first symptoms was 3 weeks to 4.5 months with an average of 1.7 months, which was considerably shorter than the treatment length.
Jaundice was the most frequently reported symptom, rarely also weakness, anorexia, nausea, vomiting, abdominal pains, dark urine, pale stools, and itching.
High serum activities are found for ALT but not for ALP, suggestive of a hepatocellular type of toxic liver injury in patients with GC hepatotoxicity.
Histology showed predominantly liver cell necrosis and hepatitis.
Outcome was favorable in all 16 patients, with lack of both acute liver failure and requirement of a liver transplant.
In one patient, good prognosis was sustained even after 7 months of continued GC Use despite presence of emerging GC hepatotoxicity.
GC hepatotoxicity usually represents the hepatocellular and idiosyncratic type of liver injury with its metabolic subgroup, characterized as acute clinical course.
The underlying mechanism(s) leading to GC hepatotoxicity as well as possible culprit(s) are still unknown.
In cases of liver disease, causality for GC was verified and creates concern regarding safety of patients and pharmacovigilance considerations.
Due to lack of epidemiologic data, the incidence of GC hepatotoxicity cannot accurately be calculated but appears to be low.

Preferred documentation: The data are based on the cases of 16 patients with GC hepatotoxicity and highly probable or probable causality levels for GC and derived from a previous report (Teschke et al., 2012a). Abbreviations: ALT, alanine aminotransferase; ALP, alkaline phosphatase; GC, Greater Celandine.

Although, clinical features are quite similar in HILI cases by traditional and modern medicine (Chau et al., 2011; Teschke et al., 2013f; Ma et al., 2014), there is one exception that relates to the hepatic sinusoidal obstruction syndrome (HSOS), formerly hepatic veno-occlusive disease (HVOD); this special liver injury is caused by pyrrolizidine alkaloids (PAs) contained in various TCM herbs, with its major diagnostic features of abdominal distension and pain, ascites, malaise, hepatomegaly, and body weight gain due to ascites and edema caused by fluid accumulation (Wang and Gao, 2014). Jaundice was most frequent with 84.8% in 100/118 cases of PA induced HSOS by Tu San Qi (*Gynura segetum*), ascites with 99.2% (121/122 cases, and hepatomegaly with 92.0% (104/113 cases) (Lin et al., 2011; Gao et al., 2012).

In a typical HILI case unrelated to PAs, the chronology of symptoms may follow a particular stepwise pattern, as described for HILI caused by Indian Ayurvedic herbs through an excellent observation by a patient under treatment for her vitiligo (Teschke and Bahre, 2009). Her symptoms started with pruritus, followed by loss of appetite, fatigue, nausea, vomiting, dark urine, light stool, until finally jaundice was recognized by her family physician; this sequence of symptoms stretched over almost 4 months under continued herbal medication.

Patients with HILI may be asymptomatic with increased values observed by chance, monosymptomatic, or polysymptomatic. Latency period describes the interval between initiation of herb use and time of onset, evidenced by emerging symptoms or increased liver values. Liver injury by herbal TCM develops slowly with clinical symptoms appearing between 1 week and 1 month (Ma et al., 2014), or up to 150 days (Chau et al., 2011); with a longer latency period of 5–260 weeks for green tea extracts (GTE) (Mazzanti et al., 2009; Teschke et al., 2014a); or 1 week–24 months for other herbs such as kava (Teschke et al., 2008a); and 28–134 days for Greater Celandine (GC) (Teschke et al., 2011a). Finally, published HILI symptoms (Chau et al., 2011; Teschke et al., 2013a; Ma et al., 2014) are similar to those of DILI (Andrade et al., 2004; Liss and Lewis, 2009).

#### Clinical course

The clinical course of HILI is variable with details provided in most publications as referenced (Tables 1, 2). For HILI cases, some details of treatment modalities by herbal products of traditional and modern medicine are provided, with focus on daily and cumulative dose, treatment duration, latency period, and reexposure duration (Table 5). With cessation of herbal use, clinical signs usually vanish along with improvements or normalization of initially increased liver values, as illustrated by few examples (Verucchi et al., 2002; Vanderperren et al., 2005; Teschke and Bahre, 2009; Furukawa et al., 2010; Valente et al., 2010; Yang et al., 2010). A well described dechallenge of liver values in suspected HILI is one of the key items to suspect causality for a particular herb. Patients with HILI caused by herbal TCM or modern herbal medicine commonly experience an acute type of liver injury, which is self-limited upon withdrawal of the offending herb with an overall good prognosis. Whether herbs may cause chronic forms of HILI has not yet been evaluated in detail (García-Cortés et al., 2008). However, persistence of increased liver values raises the question whether these are due to a preexisting liver disease present prior to herbal use rather than to HILI (Picciotti et al., 1998). The acute type of HILI rarely may progress to acute liver failure (Stadlbauer et al., 2005; Fong et al., 2010). This is a serious condition that may require a liver transplant and eventually leads to death (Perharic-Walton and Murray, 1992; Yoshida et al., 1996; Haller et al., 2002; Adachi et al., 2003; Estes et al., 2003; Yuen et al., 2006; Sohn et al., 2008; Fong et al., 2010). Between 1992 and 2008; in Seoul (Korea) alone, 24 patients underwent liver transplantation due to toxic hepatitis caused by herbal TCM (Sohn et al., 2008), causing concern in view of poorly documented efficacy of herbal TCM (Manheimer et al., 2009; Teschke, 2014).

**Table 5:** **Some chracteristics of daily and cumulative doses, treatment duration, latency period, and reexposure period of cases with hepatotoxicity by herbs of traditional and modern medicine**.

**Case**	**Sex Age**	**Herb Herbal mixture**	**Daily dose**	**Cumulative dose**	**Treatment duration**	**Latency period**	**Reexposure duration**	**References**
1.	F/62y	Aloe	420 mg	37800 mg	3.0 month	2.75 month	1.0 month	Yang et al., 2010
2.	M/71y	Chaparral	1 tablet	90 tablets	3.0 month	3.5 month	1.0 month	Batchelor et al., 1995
3.	F/39y	Chinese herbal mixture	n.a.	n.a.	2.0 month	2.0 month	0.1 month	Kane et al., 1995
4.	F/ 9y	Chinese herbal mixture	n.a.	n.a.	6.0 month	5.25 month	1.0 month	Davies et al., 1990
5.	F/66y	Chinese Jin Bu Huan	0–2 tablets	60 tablets	3.0 month	2.75 month	0.5 month	Woolf et al., 1994
6.	M/46y	Chinese Jin Bu Huan	0–3 tablets	216 tablets	6.0 month	6.0 month	1.0 month	Woolf et al., 1994
7.	F/52y	Chinese Syo Saiko To	7.5 g	338 g	1.5 month	1.5 month	1.0 month	Itoh et al., 1995
8.	F/58y	Chinese Syo Saiko To	7.5 g	675 g	3.0 month	3.0 month	0.25 month	Itoh et al., 1995
9.	F/42y	Chinese Syo Saiko To	7.5 g	158 g	0.75 month	0.75 month	0.07 month	Itoh et al., 1995
10.	F/54y	Germander	600 mg	23,400 mg	1.3 month	1.3 month	1.0 month	Larrey et al., 1992
11.	F/25y	Germander	n.a.	n.a.	4.0 month	4.0month	0.75 month	Larrey et al., 1992
12.	M/48y	Germander	900 mg	81,000 mg	3.0 month	3.75 month	0.33 month	Larrey et al., 1992
13.	F/45y	Germander	260 mg	468,00 mg	6.0 month	6.0 month	0.25 month	Laliberté and Villeneuve, 1996
14.	F/42y	Greater Celandine	600 mg	162,000 mg	9.0 month	2.0 month	1.5 month	Strahl et al., 1998
15.	F/56y	Green tea	14 ml	210 ml	4.0 month	3.3 month	1.0 month	Jimenez-Saenz and Martinez-Sanchez, 2006
16.	F/37y	Green tea	n.a.	n.a.	4.0 month	4.0 month	1.0 month	Bonkovsky, 2006
17.	F/63y	Herbalife	n.a.	n.a.	4.0 month	3.5 month	n.a.	Hoffmann et al., 2005
18.	F/39y	Kava	60 mg	10,800 mg	6.0 month	6.0.month	0.5 month	Strahl et al., 1998
19.	F/49y	Mistletoe	50 mg	1500 mg	1.0 month	1.0 month	1.0 month(?)	Harvey and Colin-Jones, 1981
20.	M/61y	*Polygonum multiflorum*	n.a.	n.a.	0.033 month	0.033 month	0.033 month	Jung et al., 2011
21.	F/26y	Senna	100 mg	12,000 mg	4.0 month	3.0 month	n.a.	Beuers et al., 1991

In all 22 hepatotoxicity cases, causality for the respective herb or herbal mixture was ascertained by positive reexposure test results based on established criteria. Unclear: ?, Adapted data from a previous report (Teschke et al., 2014b).

Cessation of herbal use is the only therapeutic approach for HILI patients. Other options including evidence based therapy for treating patients with HILI are lacking, but on a case basis treatment was reported with glycyrrhizin (Inoue et al., 2011), ursodesoxycholic acid (Jorge and Jorge, 2005; Inoue et al., 2011), or corticosteroids (Weinstein et al, 2012).

#### Hepatotoxicity criteria

**Figure 1:**
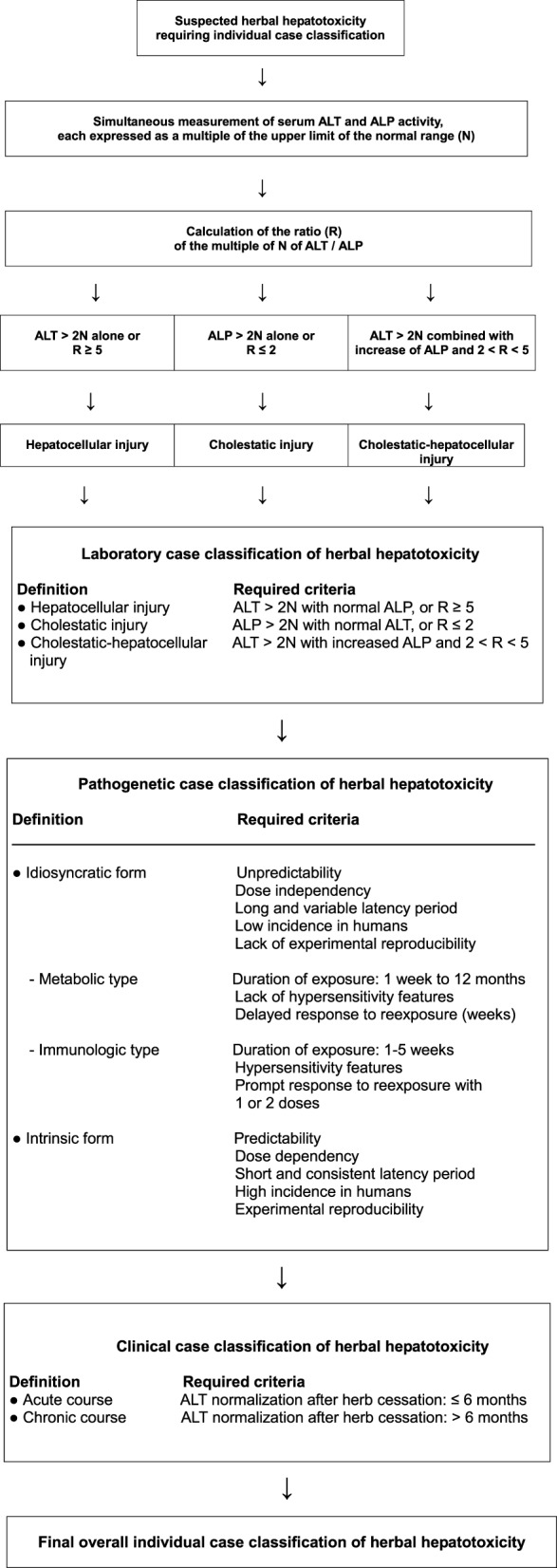
**Strategy for assessing HILI cases according to details presented previously (Teschke et al., 2013f)**. Abbreviations: ALP, alkaline phosphatase; ALT.

HILI case assessment mandates clear hepatotoxicity criteria for disease characterization including causality assignment (Teschke et al., 2013f, 2014b). Laboratory-based criteria of HILI are best defined by alanine aminotransferase (ALT) and/or alkaline phosphatise (ALP) values, expressed as N in multiples of the upper limit of their normal range (Figure 1). For ALT, recommendations initially were at >2N (Bénichou et al., 1993; Danan and Bénichou, 1993) and currently are at >5N (Björnsson et al., 2012; Teschke et al., 2014c,d) or at 3N if total bilirubin values exceed 2N (Aithal et al., 2011); for ALP, values of >2N are considered diagnostic (Bénichou et al., 1993; Danan and Bénichou, 1993; Aithal et al., 2011). Restricting the ALT criteria to >5N will eliminate unspecific ALT increases and substantiate causality at a high level of probability (Björnsson et al., 2012). Considering patients with ALT values of >2N will initially also include numerous cases with nonspecific increases, which then require thorough assessment and stringent exclusion of causes unrelated to the used herb(s). For low threshold values, the rate of alternative diagnoses is high (Teschke et al., 2013g), findings that are plausible and not unexpected (Teschke et al., 2014e). Other values such as aspartate aminotransferase (AST) are not required, unless to be used as substitute for ALT if not available.

Concern emerges whenever hepatotoxicity is assumed even if liver values were only marginally increased, not reported, or not assessed. These problems are not uncommon for cases of assumed HILI, presented for instance by the US Pharmacopeia (USP) (Mahady et al., 2008) relating to both black cohosh (BC) (Teschke, 2010c; Teschke et al., 2011d,e; Teschke and Schulze, 2012) and green tea extracts (Sarma et al., 2008; Liss and Lewis, 2009); by the WHO, relating to kava (WHO, 2007a; Teschke and Wolff, 2009); the German regulatory agency BfArM (Bundesinstitut für Arzneimittel und Medizinprodukte) (BfArM, 2002, 2005) relating to kava (Schmidt et al., 2005; Teschke et al., 2008a); or the Drug Commission of the German Medical Association (DCGMA, 2011) relating to *Pelargonium sidoides* (PS) (Teschke et al., 2012b,e). In published case reports receiving the benefit of appropriate peer reviews, the presented HILI cases commonly provide high values of aminotransferases and/or ALP and basic data support of potential hepatotoxicity, as shown also for some cases with a positive reexposure test results (Teschke et al., 2014b). In other HILI case series, however, criteria were not or incompletely documented; neglecting these aspects in effect invalidates the causality assessment.

In spontaneous reports of regulatory agencies, a clear hepatotoxicity definition was provided by EMA (2007) but not by the U.S. Pharmacopeia (USP) (Mahady et al., 2008; Sarma et al., 2008), the German BfArM (2002), or the WHO (2007a). For instance, EMA mentions cases with assumed HILI by BC but clarifies that a causal attribution cannot be made with the required certainty in face of missing liver values (EMA, 2007). Consequently, missing regulatory hepatotoxicity definitions represent confounding variables and result in false high signal cases due to regulatory case overreporting and overdiagnosing (Teschke et al., 2013f).

For reasons of transparency and assessment of case data quality, each HILI case series should provide tabulated information of available or missing case details, as done in various reports (Teschke et al., 2011a, 2012b,e) and shown as example (Table 6) (Teschke, 2010c).

**Table 6:** **Preferred documentation as example: overview of known information regarding all 69 patients with primarily suspected but not established HILI by black cohosh (BC)**.

**Presented information**	**Cases**	**Individual cases**
Brand name	22/69	1,5,11,12,13,14,15,16,17,18,19,20,21,22,23,24,28,29,30,31,32,33
Manufacturer	12/69	1,5,11,17,18,20,28,29,30,31,32,33
Plant part	06/69	1,5,8,11,28,33
Solvent	02/69	1,11
Daily dose	11/69	1,3,5,6,7,11,12,13,16,17,18
BC drug	07/69	1,11,19,21,23,24,31
BC herbal supplement	01/69	5
BC polyherbal product	14/69	2,3,12,13,14,15,16,17,18,20,22,28,30,33
Date of BC start	20/69	1,3,4,5,6,7,8,10,11,12,13,15,18,20,21,22,27,28,29,33
Date of BC end	17/69	1,3,4,5,6,7,8,10,11,12,13,15,18,19,20,21,22
Date of symptoms	24/69	1,2,3,4,5,6,7,8,10,11,12,13,14,15,16,18,19,20,21,22,23,27,32,33
Temporal association	12/69	1,4,5,6,8,10,12,13,18,20,21,22
Time on BC	16/69	1,3,4,5,6,7,8,10,11,12,13,15,18,20,21,22
Time to onset	19/69	1,3,4,5,6,7,8,9,10,11,12,13,18,20,21,22,27,28,33
ALT value	15/69	1,2,3,4,5,6,7,8,10,11,12,13,15,23,32
ALP value	12/69	1,2,3,4,5,6,7,11,12,13,23,32
Hepatotoxicity criteria	14/69	1,2,3,4,5,6,7,8,10,11,12,13,23,32
ALT de-challenge	06/69	4,6,7,10,11,15
Biliary tract imaging	08/69	4,5,6,7,9,10,15,28
HAV	13/69	1,2,3,4,5,6,7,8,9,10,11,14,15
HBV	12/69	1,3,4,5,6,7,8,9,10,11,14,15
HCV	13/69	1,2,3,4,5,6,7,8,9,10,11,14,15
CMV	09/69	1,4,5,6,8,10,11,14,15
EBV	09/69	1,4,5,6,8,10,11,14,15
HSV	03/69	4,6,8
VZV	01/69	8
Co-medication/herbal mixture	23/69	2,3,4,5,6,7,11,12,13,14,15,16,17,18,20,22,23,27,28,29,30,32,33
BC undetermined product	13/69	4,6,7,8,9,10,25,26,27,29,32,34–69

PreferreThe group of the 69 cases consisted of 11 case reports (cases 1–11), 13 TGA cases from Australia (cases 12–24), 2 CADRMP cases from Canada (cases 25 and 26), 7 MedWatch/ FDA cases from the United States (cases 27–33), and 33 EMA cases from the European Union (cases 34–69). Details and references of the 69 cases were published earlier (Teschke et al., 2010). Abbreviations: ALP, alkaline phosphatase; ALT alanine aminotransferase; BC, black cohosh; CMV, cytomegalovirus; EBV, Epstein Barr virus; HAV, hepatitis A virus, HBV, hepatitis B virus, HCV, hepatitis C virus; HSV, herpes simplex virus; VZV, varicella zoster virus.

#### HILI case characteristics

Hepatotoxicity classification is mandatory in cases of assumed HILI to facilitate further evaluation of reexposure results and CIOMS assessments (Teschke et al., 2013f). Based on specific laboratory constellations, differentiation of the hepatocellular, cholestatic or mixed form of hepatotoxicity is feasible by comparing serum activities of ALT and ALP at the time HILI diagnosis is first suspected (Bénichou et al., 1993; Danan and Bénichou, 1993; Teschke et al., 2014d). Enzyme activity is expressed as a multiple of the upper limit of the normal range (N), and the ratio (R) of ALT/ALP is calculated. Liver injury is classified as hepatocellular, if ALT > 2N alone or *R* = 5; cholestatic, when there is an increase of ALP > 2N alone or when *R* = 2; of the mixed type if ALT > 2N, ALP is increased, and 2 < *R* < 5 (Figure 1). In a HILI case series of herbal TCM consisting of 27 patients, the pattern of liver injury was hepatocellular in 82% of the cases, cholestatic in 11%, and mixed in 7% (Chau et al., 2011).

#### Liver histology

Liver biopsy in HILI and DILI cases requires special attention in any clinical hepatology setting, balancing benefits and risks for the patient (Teschke and Frenzel, 2014). Published and spontaneous HILI reports often contain detailed histological descriptions of liver biopsy findings, mostly associated with pictures obtained by microscopy. This erroneously implies that liver biopsy is an essential part of routine case assessments (BfArM, 2002; Teschke et al., 2008a, 2011a, 2012a,b,f,g; Teschke, 2010c). Histology data were also presented by narrative HILI case reports lacking even any causality for a particular herb (Teschke et al., 2012f,g). This raises the question whether a liver biopsy is justified, considering also that there were no histological findings recognized as specific for all hepatotoxicity cases (Ramachandran and Kakar, 2009). Liver biopsy in chronic hepatotoxicity cases to define prognosis in the absence of an expected specific therapy option remains debatable (Teschke and Frenzel, 2014).

To evaluate liver histology findings, a retrospective case analysis of pathological changes in HILI selectively caused by one single herb with established causality appears the best approach. For instance, HILI cases of kava and GC have such an established causality track. In 12 GC HILI patients with a probable or highly probable causality grading for GC, prevailing histological features included hepatitis, single or confluent liver cell necrosis, inflammation, rarely fibrosis, and cholestasis (Teschke et al., 2011a, 2012a,e). In eight HILI patients with a highly probable, probable or possible causality for kava, liver histology showed hepatitis, liver cell necrosis, and rarely bile duct proliferation and intrahepatic cholestasis (Teschke et al., 2008a). Therefore, at least for the two herbs GC and kava, the histological features are quite uniform and restricted to two major features, hepatitis and liver cell necroses. These histological characteristics, however, are also found in most other liver diseases unrelated to herbs, obviating liver biopsy in suspected HILI cases due to unspecific results.

Additional insights are provided by the analysis of cases with positive reexposure tests, done unintentionally with the incriminated herb or herbal mixture, and available liver histology results. For instance, in HILI by a herbal mixture of TCM, total liver necrosis prevailed (Perharic-Walton and Murray, 1992); germander (*Teucrium chamaedrys*) caused hepatocyte necrosis with lobular inflammatory infiltration mainly by mononuclear cells, associated with slightly fibrous portal tracts containing inflammatory cells (Larrey et al., 1992); senna use resulted in liver cell necrosis around the central veins as well as portal and lobular infiltration by lymphocytes, histiocytes, and rare plasma cells (Beuers et al., 1991); chaparral intake was associated with hepatocellular necrosis combined with inflammation, portal tract expansion, mild cholestasis and fibrous septation (Batchelor et al., 1995); and the herbal TCM Chinese skullcap (*Scutellaria baicalensis*) was considered to cause acidophil bodies, ballooned hepatocytes, lobular inflammatory cell infiltrates including eosinophils, and portal tracts containing mononuclear cells and eosinophils (Yang et al., 2012a). Based on causality established by positive reexposure results, these few examples may provide some insight in morphological liver changes due to herbal use.

Histological features usually are not clinically relevant, but some clinicians still consider a liver biopsy an important part of the diagnostic work-up in suspected hepatotoxicity cases. The question is whether histological results changed the initial diagnosis or benefited the individual patient. In two cases of initially suspected HILI, however, histological findings of giant cell hepatitis were reported and completely ignored (Dunbar and Solga, 2007; Schoepfer et al., 2007), while the clinical course and this particular histological pattern best fitted with an existing severe virus infection with hepatic involvement rather than herbal hepatotoxicity (Teschke and Schwarzenboeck, 2009).

Clearly, the pathologist is not helpful offering diagnoses such as HILI or liver injury compatible with or suggestive for herbal use. Overall, liver histology as a supporting routine method for assessing HILI cases is not recommended, it commonly adds little new specific diagnostic clues as information to the case without benefit for the patient; as an invasive procedure, rare but potentially life threatening complications may occur (Teschke and Frenzel, 2014).

#### Alternative causes

**Figure 2:**
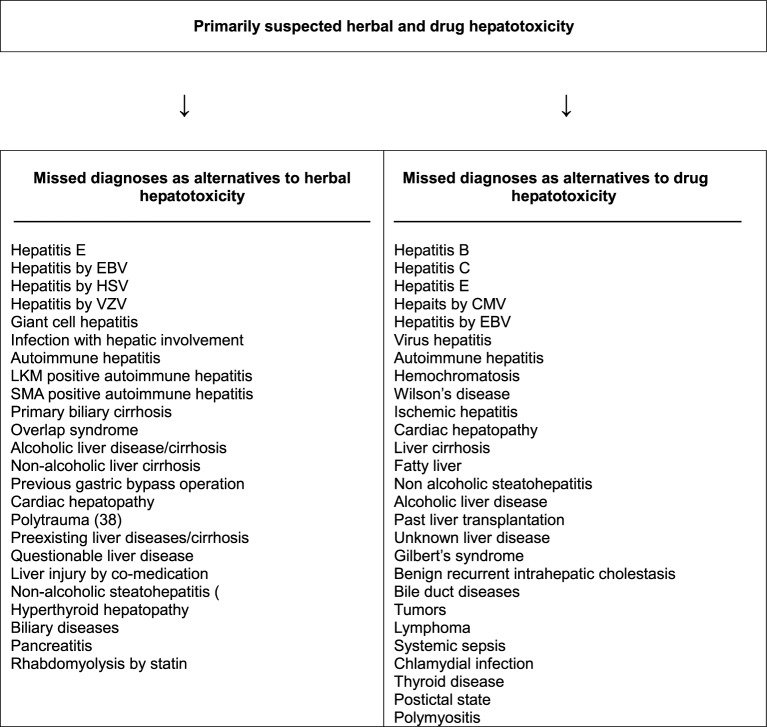
**Missed diagnoses in cases of initially suspected hepatotoxicity by herbs and synthetic drugs**. Adapted from a previous report (Teschke et al., 2013f), which provides the respective references for each item presented above. Abbreviations: CMV, cytomegalovirus; EBV, Epstein Barr virus; HSV herpes simplex virus; LKM, liver kidney microsomes; SMA, smooth muscle antibodies; VZV, varicella zoster virus.

Unrecognized alternative diseases are a real clinical problem when caring for patients with initially assumed but later not confirmed HILI. Several hundred liver diseases have to be considered as diagnoses alternative to HILI, to be ruled out under clinical aspects and with specific diagnostic tools. As a reminder for clinicians, a checklist with details for these alternative diagnoses is available (Table 7) (Teschke et al., 2014d). Numerous missed diagnoses were found upon reevaluation of initially assumed HILI cases, with similar problems for DILI (Figure 2) (Teschke et al., 2013g, 2014a). Exclusion of hepatitis E and infections by cytomegalovirus (CMV), Epstein Barr virus (EBV), herpes simplex virus (HSV), and varicella zoster virus (VZV) should be obligatory rather than facultative (Table 7).

**Table 7:** **Differential diagnoses of HILI**.

**Differential diagnosis**	**Diagnostic parameters**	**Diagnostic exclusion done for patient's assessment**		
		**Yes**	**NO**	**Partial**
Hepatitis A	Anti-HAV-IgM	□	□	□
Hepatitis B	Anti-HBc-IgM, HBV-DNA	□	□	□
Hepatitis C	Anti-HCV-IgM, HCV-RNA	□	□	□
Hepatitis E	Anti-HEV-IgM, Anti-HEV-IgG, HEV-RNA	□	□	□
Cytomegalovirus (CMV)	CMV-PCR, titre change for Anti-CMV-IgM and Anti-CMV-IgG	□	□	□
Epstein Barr virus (EBV)	EBV-PCR, titre change for Anti-EBV-IgM and Anti-EBV-IgG	□	□	□
Herpes simplex virus (HSV)	HSV-PCR, titre change for Anti-HSV-IgM and Anti-HSV- IgG	□	□	□
Varicella zoster virus (VZV)	VZV-PCR, titre change for Anti-VZV-IgM and Anti-VZV- IgG	□	□	□
Other virus infections	Specific serology of Adenovirus, Coxsackie-B-Virus, Echovirus, Measles virus, Rubella virus, Flavivirus, Arenavirus, Filovirus, Parvovirus, HIV, and others	□	□	□
Other infectious diseases	Specific assessment of bacteria, fungi, parasites, worms, and others	□	□	□
Autoimmune hepatitis (AIH) type I	Gamma globulins, ANA, SMA, AAA, SLA/LP, Anti-LSP, Anti-ASGPR	□	□	□
Autoimmune hepatitis (AIH) type II	Gamma globulins, Anti-LKM-1 (CYP 2D6), Anti-LKM-2 (CYP 2C9), Anti-LKM-3	□	□	□
Primary biliary cholangitis (PBC)	AMA, Anti PDH-E2	□	□	□
Primary sclerosing cholangitis (PSC)	p-ANCA, MRC	□	□	□
Autoimmune cholangitis (AIC)	ANA, SMA	□	□	□
Overlap syndromes	See AIH, PBC, PSC, and AIC	□	□	□
Non alcoholic steatohepatitis (NASH)	BMI, insulin resistance, hepatomegaly, echogenicity of the liver	□	□	□
Alcoholic liver disease (ALD)	Patient's history, clinical and laboratory assessment, sonography	□	□	□
Drug induced liver injury (DILI)	Patient's history, clinical and laboratory assessment, sonography, use of the CIOMS scale	□	□	□
Cocaine, ecstasy and other amphetamines	Toxin screening	□	□	□
Rare intoxications	Toxin screening for household and occupational toxins	□	□	□
Hereditary hemochromatosis	Serum ferritin, total iron-binding capacity, genotyping for C2824 and H63D mutation, hepatic iron content	□	□	□
Wilson's disease	Copper excretion (24 h urine), ceruloplasmin in serum, free copper in serum, Coombs-negative hemolytic anemia, hepatic copper content, Kayser-Fleischer-Ring, neurologic-psychiatric work-up, genotyping	□	□	□
Porphyria	Porphobilinogen in urine, total porphyrines in urine	□	□	□
α_1_—Antitrypsin deficiency	α_1_—Antitrypsin in serum	□	□	□
Biliary diseases	Clinical and laboratory assessment, hepatobiliary sonography, MRC	□	□	□
Pancreatic diseases	Clinical and laboratory assessment, sonography, CT, MRT	□	□	□
Celiac disease	TTG antibodies, endomysium antibodies, duodenal biopsy	□	□	□
Anorexia nervosa	Clinical context	□	□	□
Parenteral nutrition	Clinical context	□	□	□
Cardiopulmonary diseases	Cardiopulmonary assessment of congestive heart disease, myocardial infarction, cardiomyopathy, cardiac valvular dysfunction, pulmonary embolism, pericardial diseases, arrhythmia, hemorrhagic shock, and various other conditions	□	□	□
Addison's disease	Plasma cortisol	□	□	□
Thyroid diseases	TSH basal, T4, T3	□	□	□
Grand mal seizures	Clinical context of epileptic seizure (duration > 30 min)	□	□	□
Heat stroke	Shock, hyperthermia	□	□	□
Polytrauma	Shock, liver injury	□	□	□
Systemic diseases	Specific assessment of M. Boeck, amyloidosis, lymphoma, other malignant tumors, sepsis, and others	□	□	□
Other diseases	Clinical context	□	□	□

This tabular compilation represents an update of a previous version (Teschke et al., 2013f). AAA, Anti-actin antibodies; AMA, antimitochondrial antibodies; ANA, antinuclear antibodies; ASGPR, Asialo-glycoprotein-receptor; BMI, body mass index; CIOMS, Council for International Organizations of Medical Sciences; CT, computer tomography; CYP, cytochrome P450; DPH, pyruvate dehydrogenase; HAV, hepatitis A virus; HBc, Hepatitis B core; HBV, hepatitis B virus; HCV, hepatitis C virus; HEV, hepatitis E virus; HILI, herb induced liver injury; HIV, human immunodeficiency virus; LKM, liver kidney microsomes; LP, liver-pancreas antigen; LSP, liver specific protein; MRC, magnetic resonance cholangiography; MRT, magnetic resonance tomography; p-ANCA, perinuclear antineutrophil cytoplasmatic antibodies; PCR, polymerase chain reaction; SLA, soluble liver antigen; SMA, smooth muscle antibodies; TSH, thyroid stimulating hormone; TTG, tissue transglutaminase.

#### Causality evaluation

#### Reexposure

Establishing the diagnosis of HILI may be cumbersome in the usual clinical setting, with experts not available in place and in time. Since, convincing biomarkers for all HILI cases are lacking, the gold standard for the diagnosis of hepatotoxicity still is a positive unintentional reexposure test result, if available (Bénichou et al., 1993; Danan and Bénichou, 1993; García-Cortés et al., 2008; Chalasani and Björnsson, 2010). Details of essential criteria are based on the conclusions of an International Consensus Meeting, as referred previously (Bénichou et al., 1993). Accordingly, required data are baseline ALT levels before reexposure, designed ALTb, and reexposure ALT levels, designed ALTr. The reexposure test is positive, if ALTr = 2ALTb and ALTb is below 5N, with N as the upper limit of the normal value (Table 8). Other variations lead to uninterpretable results. Some HILI reports mentioned a positive reaction upon reexposure, and these cases were further analyzed. Not all reexposure tests could be confirmed, partially due to lack of any details; however, for numerous herbs and herbal preparations of TCM and modern medicine, valid reexposure test results confirmed causality in the assessed HILI cases (Table 9) (Teschke et al., 2014b,d); using these cases, some characteristic features of daily and cumulative doses, divergences between treatment duration and latency period, and reexposure duration are evident (Table 5).

**Table 8:** **Conditions of unintentional reexposure tests in suspected HILI cases**.

**Reexposure test result**	**Hepatocellular type of liver injury**		**Cholestatic (±hepatocellular) type of liver injury**	
	**ALTb**	**ALTr**	**ALPb**	**ALPr**
Positive	<5N	≥2ALTb	<2N	≥2ALPb
Negative	<5N	<2ALTb	<2N	<2ALPb
Negative	≥5N	≥2ALTb	≥2N	≥2ALPb
Negative	≥5N	<2ALTb	≥2N	<2ALPb
Uninterpretable	<5N	n.a.	<2N	n.a.
Uninterpretable	n.a.	≥2ALTb	n.a.	≥2ALPb
Uninterpretable	n.a.	n.a.	n.a.	n.a.

Conditions and criteria for an unintentional reexposure test, adapted from a previous report (Teschke et al., 2014b). Accordingly, required data for the hepatocellular type of liver injury are the ALT level, just before reexposure, designed as baseline ALT or ALTb, and the ALT levels during reexposure, designed as ALTr. Response to reexposure is positive, if both criteria are met: first, ALTb is below 5N with N as the upper limit of the normal value, and second ALTr ≥2ALTb. Other variations lead to negative or uninterpretable results. For the cholestatic (±hepatocellular) type of liver injury, corresponding values of ALP are to be used rather than of ALT. Abbreviations: ALP, Alkaline phosphatase; ALT, Alanine aminotransferase; n.a., not available.

**Table 9:** **Analysis of reported positive reexposure test results in cases of suspected herbal Traditional Chinese Medicine (TCM) induced liver injury**.

**Case reexposure tests in cases of suspected herbal TCM induced liver injury**
**Chinese herbal mixtures**
• 28-year old UK woman (Perharic-Walton and Murray, 1992): Chinese herbal mixture with 8 different herbs for 3–5 months. Jaundice. ALT value not available. Reexposure: episode of hepatitis reported without liver values, acute liver failure, died despite emergency liver transplantation. Both ALTb and ALTr not available → uninterpretable reexposure.
• 39-year old UK woman (Kane et al., 1995): Chinese herbal mixture with eight different herbs for 2 months. Short history of anorexia, nausea, fatigue, dark urine, yellow sclerae, jaundice. ALT 2440 U/L (normal 0–30) with R 68.3, ALT returned to normal after cessation. Reexposure after 6 weeks: ALT 1314 U/L. ALTb < 5N and ALTr = 2 ALTb → positive reexposure.
• 9-year old UK girl (Allen and Parkinson, 1990): Unclassified Chinese herbal medicine for 6 months. Nausea, anorexia, central abdominal pain, jaundice, pale stool for the past 4–21 days. ALT 1950 U/L (normal < 45) with R 13.1, ALT returned to 50 U/L after cessation. Intentional reexposure: ALT 315 U/L. ALTb < 5N and ALTr = 2ALTb → positive reexposure.
**Ho Shou Wu**
• 54-year old Korean woman (Bae et al., 2010): Unknown dose of Ho Shou Wu containing *Polygonum multiflorum* for one month. Diagnosis of toxic hepatitis. Cessation of Ho Shou Wu improved her condition. Reexposure started immediately after discharge with aggravation of liver values. English abstract and Korean article → uninterpretable reexposure.
**Huan Qin**
• 78-year old US woman (Yang et al., 2012a): Move Free Advanced® two tablets/day containing Huan Qin (*Scutellaria baicalensis*, Chinese skullcap), black catechu, glucosamine, and chondroitin for 3 weeks. Jaundice. ALT 1626 U/L (normal < 60) with R 10.2, ALT 678 U/L two weeks after cessation. Reexposure: ALT 1206 U/L. ALTb = 5N and ALTr < 2ALTb → negative reexposure.
**Hwang Geun Cho**
• 37-year old male patient from Korea (Kang et al., 2009): Hwang Geun Cho containing *Corydalis speciosa*. Jaundice. ALT 531 U/L with subsequent decline after cessation of the herb down to 146 U/ L. Unintentional reexposure two months after discharge: ALT 381 U/L. ALTb < 5N and ALTr = 2ALTb → positive reexposure.
**Ji Xue Cao**
• 61-year old Argentinian woman (Jorge and Jorge, 2005): Ji Xue Cao (*Centella asiatica*, syn.Gotu Kola) tablets for 30 days. Jaundice. ALT 1193 U/L and 2 months after cessation 18 U/L. Unintentional reexposure 7 months later: ALT 481 U/L. ALTb < 5N and ALTr = 2ALTb → positive reexposure.
• 52-year old female patient from Argentinia (Jorge and Jorge, 2005): Ji Xue Coa (*Centella asiatica*) for 6 months. Jaundice. Not further quantified elevated hepatic enzymes at beginning and after cessation. Unintentional reexposure 1 year later: ALT 1694 U/L. ALTb not availabe → uninterpretable reexposure.
**Jin Bu Huan**
• 66-year old US woman (Woolf et al., 1994): Jin Bu Huan 2 tablets at night two to three times a week for twelve weeks. Fever, nausea, fatigue for the past 5 weeks. ALT 782 U/L (normal < 35) with R 8.7, ALT declined to 47 U/L following cessation. Reexposure: ALT 941 U/L. ALTb < 5N and ALTr = 2 ALTb → positive reexposure.
• 46-year old US man (Woolf et al., 1994): Jin Bu Huan three tablets three times a week intermittently for 6 months. Fever, headaches, fatigue, tender hepatomegaly. ALT 394 U/L (normal < 35) 2 weeks after cessation with R 24.2, ALT subsequently 48 U/L. Reexposure: ALT 100 U/L. ALTb < 5 N and ALTr = 2 ALTb → positive reexposure.
• 50-year old US woman (Horowitz et al., 1996): Jin Bu Huan two to three tablets daily or intermittently for around 24 days. Fever. ALT 830 U/L and 330 U/L after cessation. Reexposure: ALT 540 U/L. ALTb = 5N and ALTr < 2ALTb → negative reexposure.
• 70-year old US woman (Horowitz et al., 1996): Jin Bu Huan three to four tablets at night three to five times a week for 31 days. Chills and fever 12 days after start of use, subsequently low-grade fever, malaise. ALT 408 U/L initially, 263 U/L after 2- week cessation, 67 U/L after 6-week cessation. Reexposure after 1 month: ALT 77 U/L. ALTb < 5N but ALTr < 2ALTb → negative reexposure.
**Lu Cha**
• 56-year old French woman (Peyrin-Biroulet et al., 2004): Mincifit® 14 ml/day containing green tea (*Camellia sinensis*, TCM Lu Cha) and *Cassia* sp. extracts for 15 days. Jaundice. ALT 54N with R 54.0, ALT normalization 2 months after cessation. Reexposure 5 years later with Dynasvelte forte® 8–12 g/day for 21 days (Green tea, *Coffea Arabica*, and chromium): ALT 99N. ALTb < 5N and ALTr = 2ALTb → positive reexposure.
• 45-year old Spanish man (Jimenez-Saenz and Martinez-Sanchez, 2006): Green tea infusion (6 cups/day) over 4 months. Asthenia and jaundice of ten days duration prior to cessation. ALT 1613 U/L (normal < 40) with R 4.3, ALT normalized within 2 months of cessation. Reexposure 6 weeks later: ALT 1460 U/L after 1 month of reuse. ALTb < 5N and ALTr = 2ALTb → positive reexposure.
• 37-year old Hispanic woman from the US (Bonkovsky, 2006): Green tea-containing product with various other herbal extracts for 4 months. Jaundice. ALT 1788 U/L (normal < 40) with R 21.7, ALT 92 U/L after withdrawal. Reexposure 1 year later for 1 month: ALT 1131 U/L. ALTb < 5N and ALTr = 2ALTb → positive reexposure.
• 23-year old Spanish woman García-Cortés et al., 2008: Green tea (*Camellia sinensis*) for 21 days. Jaundice after 19 days. ALT 56.9N with R 34.7, ALT 0.35N 3 months after withdrawal. Reexposure: ALT values not available. ALTb < 5N but ALTr not available → uninterpretable reexposure.
• 26-year old Spanish woman García-Cortés et al., 2008: Green tea for 121 days. Jaundice. ALT 32.1N with R 42.2, ALT dechallenge values not available. Reexposure: ALT values not available. Both ALTb and ALTr not available ? uninterpretable reexposure.
• 38-year old French woman (Sarma et al., 2008): Green tea (six caps Tealine®/day, containing also white and red tea) for 20 days. Symptoms not reported. ALT values not available. Reexposure: ALT value not available. Both ALTb and ALTr not available → uninterpretable reexposure.
**Ma Huang**
• 33-year old US woman (Nadir et al., 1996): Unknown daily dose of Ma Huang for around 4 weeks. Nausea, vomiting, abdominal discomfort after use for several days, jaundice under continuing Ma Huang use for another three weeks. ALT 832 U/L (normal < 65) with R 9.8. ALT dechallenge values not available. Intentional reexposure with a single dose 1 week after discharge: ALT 1586 U/L. Both ALTb and ALTr not available ? uninterpretable reexposure.
***Polygonum multiflorum***
• 61-year old Korean man (Jung et al., 2011): Unknown dose of *Polygonum multiflorum* Thunb for 1 day. Myalgia. ALT 818 U/L with R 21.6, 180 U/L after 9 days of cessation and ALTb < 5N as likely assumed. Reexposure after 11.5 months with a single dose of *P. multiflorum* Thunb: ALT 1520 U/L. ALTb < 5N and ALTr = 2ALTb → positive reexposure.
**Shou Wu Pian**
• 5-year old Netherland girl (Panis et al., 2005): Shou Wu Pian (three tablets daily) for four months. Jaundice. ALT 1543 U/L (normal < 39 U/L), 5 weeks after cessation 50 U/L. Reexposure with tablets Shou Wu Pian daily for 1 month: ALT 1277 U/L. ALTb < 5N and ALTr = 2ALTb → positive reexposure.
**Xiao Chai Hu Tang**
• 51-year old Japanese woman (Itoh et al., 1995): 7.5 g of Xia Chai Hu Tang daily for 7 weeks. Jaundice, with preexisting mild elevations of aminotransferases. ALT 855 U/L (normal < 35) with R 35.9, ALT decrease to 139 U/L upon cessation. Reexposure: ALT 186 U/L. ALTb < 5N but ALTr < 2 ALTb → uninterpretable reexposure.
• 52-year old Japanese woman (Itoh et al., 1995): Xia Chai Hu Tang 7.5 g daily for 6 weeks. Jaundice, preexistent ALT activity of 180 U/L (normal < 35). ALT 600 U/L, near normal 2.5 months after withdrawal. Reexposure: ALT 162 U/L. ALTb < 5N and ALTr = 2ALTb → positive reexposure.
• 58-year old Japanese woman (Itoh et al., 1995): Xia Chai Hu Tang 7.5 g daily for 3 months. Symptoms not reported. ALT 246 U/L (normal < 35) with R 5.0, ALT fell to near normal after 2 months of withdrawal. Intentional 7-day reexposure: ALT 265 U/L. ALTb < 5N and ALTr = 2ALT → positive reexposure.
• 42-year old Japanese woman (Itoh et al., 1995): Xia Chai Hu Tang 7.5 g daily for an unspecified time period to treat hepatitis A infection. Symptoms not specified, ALT 2 165 U/L (normal < 35) initially dropped with treatment to 42 U/L and increased 3 weeks after initiation of treatment. ALT 1335 U/L with normalization within 2 months after withdrawal. Intentional 2-day reexposure: ALT 195 U/L ALTb < 5N and ALTr = 2ALTb → positive reexposure.

Compilation of some clinical details and laboratory values for assessment of reported positive reexposure test results in 25 cases with suspected herbal hepatotoxicity by TCM products. Data are derived from previous reports, which may provide additional details (Teschke, 2014; Teschke et al., 2014b). Unless otherwise stated, reexposure was commonly unintentional. Criteria for a positive reexposure test result were used as described in Table 4, restricted to criteria provided for the hepatocellular type of liver injury. Accordingly, essential data are the ALT levels at baseline before reexposure (ALTb), and the ALT levels during reexposure (ALTr). Response to reexposure is positive if ALTr = 2ALTb and ALTb < 5N, with N as the upper limit of the normal value. Other combinations lead to negative or uninterpretable results. Serum enzyme activities were provided in U/L or multiples of N. Details for calculation of the R value are presented before (Figure 1). Abbreviation: ALT, alanine aminotransferase; AST, aspartate aminotranferase; N, upper limit of normal; R, ratio; TCM, Traditional Chinese Medicine.

#### CIOMS/RUCAM

Physicians at armlength from the patient with HILI are well advised to consider a pragmatic thorough clinical evaluation in connection with a prospective structured approach assessing causality, providing the diagnosis in time while the disease is unfolding and without delay due to waiting periods for expert rounds' conclusions months thereafter; this is a crucial issue worldwide. We clearly prefer the CIOMS scale (Council for International Organizations of Medical Sciences), also called RUCAM (Roussel Uclaf Causality Assessment Method), in its original form (Bénichou et al., 1993; Danan and Bénichou, 1993) or better its update (Tables 10, 11) (Teschke et al., 2013a, 2014d). Discussions focused on strengths and weaknesses of CIOMS, a learning system and not immutable (Andrade et al., 2004; Rochon et al., 2008; Aithal et al., 2011; García-Cortés et al., 2011; Teschke and Wolff, 2011; Teschke and Schulze, 2012; Teschke et al., 2008c, 2013e, 2014d; Lewis, 2014; NIH, 2014b). Outlined suggestions for improvement and refinement are incorporated in the updated CIOMS scale (Tables 10, 11) (Teschke et al., 2013e, 2014d). Included is now the search for additional competing causes such as sepsis; or autoimmune hepatitis, chronic hepatitis B and C, primary biliary cholangitis and sclerosing cholangitis, and genetic liver diseases. HBsAg and HBV-DNA quantification were added to distinguish HBV infection from immunization, as was HCV-RNA to correctly assess HCV infections. Specific diagnostic criteria now include PCR detection and titer changes of the respective antibodies (IgM, IgG) for CMV, EBV, HEV, HSV, and VZV infections. Hepatobiliary sonography was supplemented by color Doppler sonography including assessments of the liver vessels, endosonography, computed tomography (CT), and magnetic resonance cholangiography (MRC. Alcohol as risk factor is now specified by an intake of >2 drinks per day (>14 units/week) in woman and >3 drinks per day (21 units/week) in men, whereby one drink corresponds to 10 g ethanol. For comparison and method validation, causality has been evaluated in 101 hepatotoxicity cases by both the original and updated CIOMS scales, with identical causality results published in 6 studies (Teschke et al., 2014d). Therefore, the updated CIOMS scale was validated, and there is no need for further validation of the updated CIOMS scale vs. the original CIOMS scale.

**Table 10:**
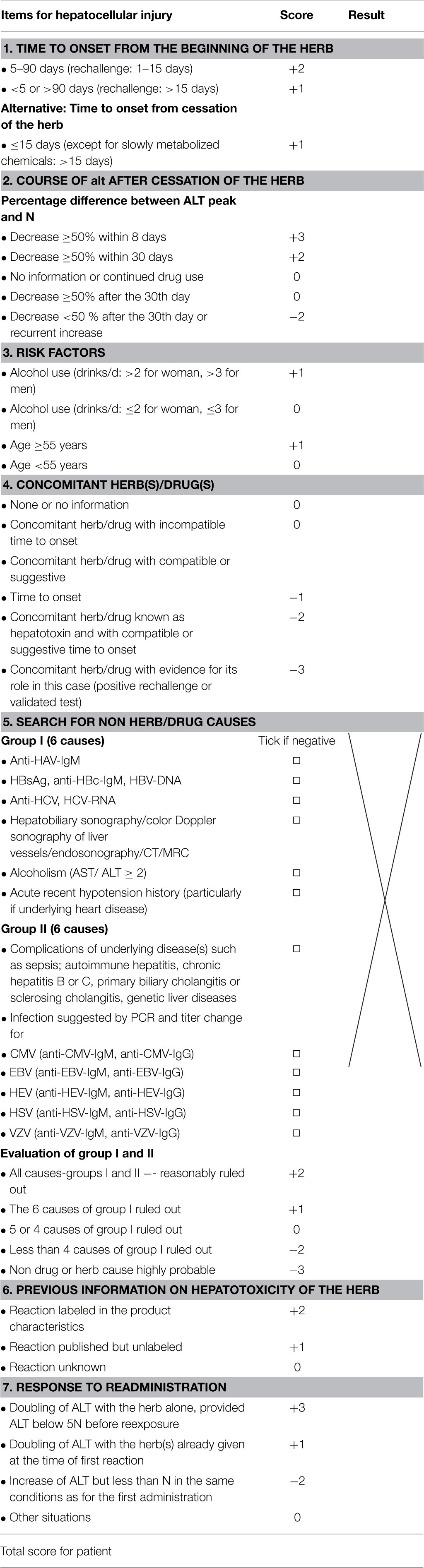
**CIOMS scale for the hepatocellular type of injury in HILI cases**.

The updated CIOMS scale is derived and modified from a previous report (Teschke et al., 2014c). The above items specifically refer to the hepatocellular type of injury rather than to the cholestatic or mixed type (shown in Table 11). Abbreviations: ALT, Alanine aminotransferase; AST, Aspartate aminotransferase; CIOMS, Council for International Organizations of Medical Sciences; CMV, Cytomegalovirus; CT, Computer tomography; DILI, Drug induced liver injury; EBV, Epstein Barr virus; HAV, Hepatitis A virus; HBc, Hepatitis B core; HBsAg, Hepatitis B antigen; HBV, Hepatitis B virus; HCV, Hepatitis C virus; HEV, Hepatitis E virus; HILI, Herb induced liver injury; HSV, Herpes simplex virus; MRC, Magnetic resonance cholangiography; N, upper limit of the normal range; VZV, Varicella zoster virus. Total score and resulting causality grading: = 0, excluded; 1–2, unlikely; 3–5, possible; 6–8, probable; ≥9, highly probable.

**Table 11:**
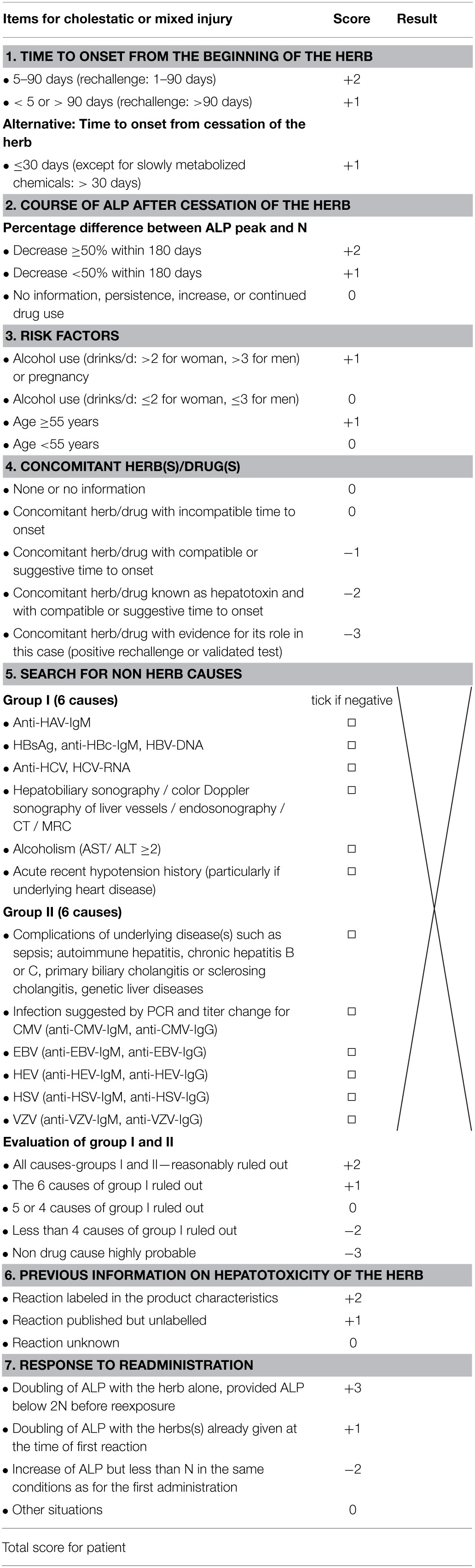
**CIOMS scale for the cholestatic or mixed type of injury in HILI cases**.

For details see legend to Table 10.

A selective compilation shows that numerous international registries and regulatory agencies as well as associated groups actually apply CIOMS for HILI and DILI cases (Table 12), with its advantages over other causality assessing approaches (Teschke et al., 2013a) including the method of DILIN (Drug-Induced Liver Injury Network) (Table 13). As opposed to the DILIN method, CIOMS fascinates by its stringent scoring system (Table 13), with its generation of a quantitative assessment (score) to address probability, which is more precise than a simple yes or no (Gunawan and Kaplowitz, 2004). Support for CIOMS was actually provided by Navarro as DILIN member and senior author of a HDS case series by applying CIOMS for causality assessment (Halegoua-De Marzio et al., 2013), whereas CIOMS was not in favor of Lewis, another DILIN member (Lewis, 2014). Although, connected with an actual commentary on a single HILI case report that did not undergo any formal causality assessment, Navarro also correctly acknowledged that CIOMS/RUCAM is the most frequently referenced scoring system (Fenkel and Navarro, 2011). This assumption supports earlier systematic analyses of 2008 (Tajiri and Shimizu, 2008). Of 61 DILI reports that were reviewed in the PubMed database over the last decade, showing that CIOMS was the most used scale. In a recent confirmative study of 573 HILI cases (Table 14), CIOMS again was the most used method applied in 275 cases (48.0%) (Teschke et al., 2013g), in line with mainstream opinion (Tables 12, 14) (Wai, 2006; Aithal et al., 2011; NIH, 2014a,b; Björnsson et al., 2012, 2013. The CIOMS scale was widely used for hepatotoxicity assessments in epidemiological studies, clinical trials, case reports, case series, regulatory analyses, and genotyping studies, as referenced in detail recently (Teschke et al., 2013f).

**Table 12:** **Selective compilation of international registries and regulatory agencies, and associated groups applying the CIOMS scale for causality evaluation in suspected HILI and DILI cases**.

**Cases**	**Products (*n*)**	**Country/Region**	**Group/Agency**	**References**
DILI	Synthetic drugs	Spain Europe	Spanish Group for the Study of the Drug-Induced Liver Disease, Malaga	Andrade et al., 2004
DILI	Synthetic drugs (461)	Spain Europe	Spanish Group for the Study of the Drug-Induced Liver Disease, Malaga	Andrade et al., 2005
DILI	Synthetic drugs (28)	Spain Europe	Spain Hepatotoxicity Registry, Grupo de Estudio Para las Hepatopatías Asociadas a Medicamentos, Malaga	Andrade et al., 2004
HILI DILI	Various herbal TCM (15) Synthetic drugs (19)	Singapore Asia	National University of Singapore	Wai, 2006
HILI	Black cohosh (31)	Various countries Europe	European Medicines Agency	EMA, 2007
HILI	Herbs (13)	Spain Europe	Spanish Liver Toxicity Registry	García-Cortés et al., 2008
HILI	Various herbal TCM (159)	Korea Asia	Chungnam National University, Daejeon	Kang et al., 2008
DILI	Synthetic drugs (80)	Serbia Europe	Medicines and Medical Devices Agency of Serbia, Belgrade	Miljkovic et al., 2011
HILI	Herbal *Polygonum Multiflorum* (25)	Korea Asia	Gyeongsang National University School of Medicine, Jinju/Sungkyunkwan University School of Medicine, Changwon	Jung et al., 2011
HILI	Various herbal TCM (27)	Hong Kong	Hong Kong Herb-Induced Liver Injury Network (HK-HILIN), Hong Kong	Chau et al., 2011
DILI	Statins (73)	Iceland/Sweden Europe	National University Hospital Reykjvik/ University of Gothenburg/ Swedish Adverse Drug Reactions Advisory Committee (SADRAC)	Björnsson et al., 2012
DILI	Various synthetic Drugs	Spain Latin America	Spanish-Latin American Network on drug induced liver Injury, in progress	Bessone, 2012
HILI	Some Herbalife® products	Various countries	Various groups	Halegoua-De Marzio et al., 2013
HILI	Various herbal and dietary supplements (HDS)	Spain Europe	Spanish group for the Study of the Drug-Induced Liver Injury	Robles-Diaz et al., 2015

**Table 13:** **Core elements of the updated CIOMS scale vs. DILIN method**.

**Items**	**CIOMS scale**	**DILIN method**
Accurate time frame of latency period (score)	+	0
Detailed time frame of challenge (score)	+	0
Clear time frame of dechallenge (score)	+	0
Recurrent ALT or ALP increase (score)	+	0
Definition of risk factors (score)	+	0
Details to exclude alternative diagnoses (score)	+	0
Assessment of HAV, HBV, HCV, HEV (score)	+	0
Assessment of CMV, EBV, HSV, VZV (score)	+	0
Liver and biliary tract imaging (score)	+	0
Color Doppler sonography of liver vessels (score)	+	0
Assessment of preexisting diseases (score)	+	0
Evaluation of cardiac hepatopathy (score)	+	0
Individual score of alternative diagnoses (score)	+	0
Qualified score of individual comedication (score)	+	0
Prior known hepatotoxicity of the herb (score)	+	0
Search for unintended reexposure (score)	+	0
Definition of unintended reexposure (score)	+	0
Qualified criteria of unintended reexposure (score)	+	0
Laboratory criteria for hepatotoxicity	+	+
Laboratory hepatotoxicity pattern	+	+
Hepatotoxicity specific method	+	+
Structured, liver related method	+	0
Quantitative, liver related method	+	0
Validated method for hepatotoxicity (gold standard)	+	0

Adapted and derived from a previous report (Teschke et al., 2013a). Latency period indicates time from herb start to symptoms, alternatively to abnormal liver tests. The symbol + shows that this item is present and the symbol 0 indicates lack of this item. Abbreviations: ALT, Alanine aminotransferase; ALP, Alkaline phosphatase; CIOMS, Council for International Organizations of Medical Sciences; CMV, cytomegalovirus; DILIN, Drug-Induced Liver Injury Network;EBV, Epstein Barr virus; HAV, Hepatitis A virus; HBV, Hepatitis B virus; HCV, Hepatitis C virus; HEV, Hepatitis E virus; HSV, Herpes simplex virus; VZV, Varicella zoster virus.

**Table 14:** **Compilation of causality assessment methods used in suspected HILI cases**.

**Herbs Herbal products**	***Ad hoc* (*n*)**	**WHO (*n*)**	**CIOMS (*n*)**	**Naranjo (*n*)**	**DILIN (*n*)**	**KL (*n*)**	**References**
Kava	20						BfArM, 2002
Kava		30					Denham et al., 2002
Kava	20						Teschke et al., 2003
Kava			36				Stickel et al., 2003
Kava		80					Schmidt et al., 2005
Greater Celandine	23						BfArM, 2005
Black cohosh			31				EMA, 2007
Herbalife products		12					Elinav et al., 2007
Herbalife products		12					Schoepfer et al., 2007
Kava			26				Teschke et al., 2008a
Black cohosh				30			Mahady et al., 2008
Green tea				34			Sarma et al., 2008^[^
Black cohosh			4				Teschke and Schwarzenboeck, 2009
Black cohosh			9				Teschke et al., 2009
Kava			31				Teschke, 2010a
Hydroxycut					17		Fong et al., 2010
Black cohosh			22				Teschke et al., 2011e
Greater Celandine			22				Teschke et al., 2011a
Herbalife products						20	Manso et al., 2011
Various herbs			45				Chau et al., 2011
Greater Celandine			21				Teschke et al., 2012e
*Pelargonium sidoides*			15				Teschke et al., 2012c
*Pelargonium sidoides*			13				Teschke et al., 2012d
Sum (n)	63	134	275	64	17	20	
Sum (percent)	11.0%	23.4%	48.0%	11.2%	3.0%	3.4%	

The data are derived from a study evaluating alternative causes in suspected HILI cases (n = 573) (Teschke et al., 2013g). For the 275 CIOMS cases, causality assessment was performed with the updated CIOMS scale, the original CIOMS scale, or early CIOMS version. Abbreviations: Ad hoc, ad hoc approach; CIOMS, Council for International Organizations of Medical Sciences scale; DILIN, Drug Induced Liver Injury Network method; KL, Karch & Lasagna method; Naranjo, Naranjo scale; WHO, World Health Organization method.

CIOMS is structured, quantitative, and specific and validated for hepatotoxicity, and considers all its core elements (Tables 10, 11) (Teschke et al., 2014d). It was developed by an international expert panel and validated by cases with positive reexposure tests as gold standard, showing good sensitivity (86%), specificity (89%), positive predictive value (93%), and negative predictive value (78%) (Bénichou et al., 1993). Of note, the scales for the hepatocellular and the cholestatic (± hepatocellular) type of injury differ slightly (Tables 10, 11).

The CIOMS scale was conceptualized and developed in consensus meetings organized at the request of the Council for International Organizations of Medial Sciences (CIOMS) (Bénichou et al., 1993; Danan and Bénichou, 1993), aiming to overcome experts' previous problems with unstructured and unquantified evaluations lacking defined and scored items,resulting in debated causality assignments. This CIOMS scale represented a breakthrough in DILI and HILI causality assessment methods and extended, specified, and quantified preceding versions (Danan, 1988; Bénichou, 1990). The basis for CIOMS was provided by eight experts in hepatology from 6 countries and included J. P. Benhamou (France), J. Bircher (Germany), G. Danan (France), W. C. Maddrey (USA), J. Neuberger (UK), F. Orlandi (Italy), N. Tygstrup (Denmark), and H. J. Zimmerman (USA) (Bénichou et al., 1993; Danan and Bénichou, 1993). These experts in the field evaluated DILI cases for case characteristics, hepatotoxicity criteria, liver injury pattern, and reexposure criteria; they standardized DILI case assessment with specific, quantitative items and validated their method with established positive reexposure DILI case results (Bénichou et al., 1993; Danan and Bénichou, 1993). CIOMS was developed for assessment of a single drug containing a synthetic product and may be used for a single herb containing multiple chemical constituents, but does not allow causality attribution to a single constituent.

CIOMS provides a differentiating range of causality grades for the responsible agent(s) and clearly delineates liver specific criteria for challenge, dechallenge, exclusion of unrelated diseases, and comedication (Bénichou et al., 1993; Danan and Bénichou, 1993). It even takes into account atypical chronology with +1 point for challenge periods of <5 days or > 90 days, whereas the period of 5–90 days renders +2 points (Table 10). It is well adapted for cases with missing data. Physicians suspecting herbal hepatotoxicity can easily use CIOMS, results are readily available within a few minutes.

To facilitate valid actual assessment and possible external reevaluation, CIOMS scale data should be provided individually point by point for each patient (Tables 10, 11), along with the list of alternative diagnoses to be excluded (Table 7). In publications of HILI cases and for submission to regulatory agencies as spontaneous reports, the completed CIOMS scale with all items including individual and final scores should be supplied to ensure data transparency, as published before (Teschke et al., 2008a, 2011a,d,e, 2012b,d,h; Teschke and Bahre, 2009; Teschke, 2010a,c) and presented as example, using the CIOMS scale of the hepatocellular type of injury (Table 15).

**Table 15:** **Preferred documentation as example: Tabulated causality assessment of 15 patients with primarily suspected HILI by *Pelargonium sidoides* (PS)**.

**Items**	**Score**	**Patients 1–15**														
		**1**	**2**	**3**	**4**	**5**	**6**	**7**	**8**	**9**	**10**	**11**	**12**	**13**	**14**	**15**
**1. TIME TO ONSET FROM THE BEGINNING OF THE HERB**																
• 5–90 days	+2	?	+2		+2		+2		+2	+2		+2	+2	?	?	
• <5 or >90 days	+1	?		+1		+1		+1			+1			?	?	+1
**2. TIME TO ONSET FROM CESSATION OF THE HERB**																
• ≥15 days	+1	?	+1	+1	+1	+1	+1	+1	+1	+1	?	+1	+1	?	?	+1
**3. COURSE OF alt AFTER CESSATION OF THE HERB**																
• Decrease ≥50% within 8 days	+3				+3								+3			
• Decrease ≥50% within 30 days	+2									+2						+2
• No information	0	0	0	0			0	0	0		0	0		0	0	
• Decrease ≥50% after the 30th day	0															
• Decrease <50% after the 30th day or recurrent increase	−2					?										
**4. RISK FACTOR ETHANOL**																
• Alcohol use (drinks/d: >2 for woman, >3 for men)	+1			?											?	
• No alcohol use (drinks/d: ≤2 for woman, ≤3 for men)	0	0	0		0	0	0		0	0	0	0	0	0	?	0
**5. RISK FACTOR AGE**																
• ≥55 years	+1	+1						+1							+1	
• <55 years	0		0	0	0	0	0		0	0	0	0	0	0		0
**6. CONCOMITANT HERB(S)/DRUG(S)**																
• None or no information	0	0	0			0	0	0		0		0	0	0		
• Concomitant herb/drug with incompatible time to onset	0										0					
• Concomitant herb/drug with compatible or suggestive time to onset	−1				−1											
• Concomitant herb/drug known as hepatotoxin and with compatible or suggestive time to onset	−2			−2					−2						?	−2
• Concomitant herb/drug with evidence for is role in this case (positive rechallenge or validated test)	−3															
**7. SEARCH FOR NON HERB/DRUG CAUSES**																
**Group i (6 causes)**																
• Anti-HAV-IgM							–			–	–					–
• Anti-HBc-IgM/HBV-DNA							–			–	–					–
• Anti-HCV-IgM/HCV-RNA							–			–	–					–
• Hepato-biliary sonography/color Doppler sonography of liver vessels							–			–						–
• Alcoholism (AST/ ALT ≥2)		–		?		–	–	–	–	–	–	–	–	–		–
• Acute recent hypotension history (particularly if underlying heart disease)		–			–	–	–	–	–	–	–	–	–			–
**Group II**																
• Complications of underlying disease(s) such as sepsis; or: autoimmune hepatitis, chronic hepatitis B and C, primary biliary cholangitis and sclerosing cholangitis, genetic liver diseases		?			–	?	+		–	+	+	+	–	+		–
• Infection suggested by PCR and titre change for																
CMV (Anti-CMV-IgM/IgG)										–						
EBV (Anti-EBV-IgM/IgG)										–						
HSV (Anti-HSV-IgM/IgG)																
VZV (Anti-VZV-IgM/IgG)																
**Evaluation of group I and II**																
• All causes—group I and II—reasonably ruled out	+2															
• The 6 causes of group I ruled out	+1															
• 5 or 4 causes of group I ruled out	0															
• Less than 4 causes of group I ruled out	−2				−2				−2							
• Non drug cause highly probable	−3	−3	−3	−3		−3	−3	−3		−3	−3	−3	−3	−3	−3	−3
**8. PREVIOUS INFORMATION ON HEPATOTOXICITY OF THE HERB**																
• Reaction labeled in the product characteristics	+2	+2	+2	+2	+2	+2	+2	+2	+2	+2	+2	+2	+2	+2	+2	+2
• Reaction published but unlabelled	+1															
• Reaction unknown	0															
**9. RESPONSE TO READMINISTRATION**																
• Doubling of ALT with the herb alone	+3															
• Doubling of ALT with the herbs/drugs already given at the time of first reaction	+1															
• Increase of ALT but less than N in the same conditions as for the first administration	−2															
• Other situations	0															
• Total points for patient		0	+2	−1	+5	+1	+2	+2	+1	+4	0	+2	+5	−1	+1	+1

Preferred documentation of causality assessment using the scale of the updated CIOMS (Council for International Organizations of Medical Sciences), considering the items for the hepatocellular type of liver injury and the data of a previous report of 15 patients with primarily suspected HILI by Pelargonium sidoides (PS) (Teschke et al., 2012b). In the above section 6 of concomitant herb(s)/drug(s), the following products were considered: synthetic drugs, dietary supplements including herbal ones, and polyherbal products. In the section 7 (search for non-herb/drug causes), the symbol of—denotes that the obtained result was negative and that of + was positive, whereas lack of a symbol indicates that assessment was not performed. ALT denotes alanine aminotransferase, AST aspartate aminotransferase, HAV hepatitis A virus, HBc hepatitis B core, HBV hepatitis B virus, HCV hepatitis C virus, CMV cytomegalovirus, EBV Epstein Barr virus, HSV herpes simplex virus, PCR Polymerase Chain Reaction, VZV varicella zoster virus. Total points/causality: ≤0/excluded; 1–2/unlikely; 3–5/possible; 6–8/probable; ≥9/highly probable.

Thus, we strongly recommend for HILI case assessment a sequential approach, starting with thorough clinical evaluations and concomitant prospective causality evaluation by the updated CIOMS scale (Tables 10, 11), followed by optional expert opinion based on scored CIOMS items, if uncertainty remains, and finally for reasons of transparency, appropriate documentation of case details (Tables 3, 6, 7, 15).

#### DILIN method

With use restricted to its own country, the United States DILIN causality method (Chalasani et al., 2008) does not operate within the international HILI and DILI mainstream domains, as opposed to CIOMS (Table 12). The DILIN method may create problems even in its homeland when physicians are waiting for conclusions of expert circles at times HILI is unfolding. Representing a post-clinical, postponed evaluation rather than a rapid assessment of HILI as a critical disease, the DILIN method will not gain the same international popularity as its counterpart CIOMS (Tables 9, 10, 12), also due to other major shortcomings (Table 13).

Although, various CIOMS criteria (Danan and Bénichou, 1993) have been incorporated in the DILIN method (Rochon et al., 2008), the DILIN group missed the chance to establish the fundaments of a newly conceptualized causality method for DILI and HILI, considering the well known pros ad cons of CIOMS (García-Cortés et al., 2011; Teschke et al., 2013a, 2014d), rather than commenting on few shortcomings of CIOMS at the expense on its own DILIN method (Lewis, 2014). As opposed to the transparent CIOMS results shown by tables with individual scored items (Tables 10, 11) and the applied scale with actual 15 assumed HILI cases (Table 15), the DILIN method lacks both transparency and individual item scorings (Teschke et al., 2013a, 2014d). Results presented as percentage ranges only and not given as clearly defined, individually scored items before are irrelevant. Proving a moderate reliability of their DILIN causality approach is far from concordance (Rochon et al., 2008; Teschke et al., 2014d); expert opinion validation therefore seems to be irrelevant. DILIN should close the present evaluating gap by recommending physicians in clinical practice to use CIOMS a priori to improve causality assessment already at origin of clinical data, while DILIN may later use these CIOMS-based itemized data for own assessment.

#### Naranjo scale

Causality assessment of hepatotoxicity cases by the Naranjo scale (Naranjo et al., 1981) with its known shortcomings is problematic (Table 13), although favored by the United States Pharmacopeia (USP) (Mahady et al., 2008) but rejected by the United States DILIN group (Lewis, 2014) and other groups (García-Cortés et al., 2011). This scale relates toxic drug reactions to general pharmacological drug actions rather than specifically to idiosyncratic reactions like hepatotoxicity; it contains drug concentrations and monitoring, dose relationship including decreasing dose, placebo response, cross-reactivity, and confirmation of the ADR using unidentified objective evidence, which are irrelevant for HILI (Naranjo et al., 1981; Teschke and Wolff, 2011; Teschke and Schulze, 2012). The hepatotoxicity unspecific feature of the Naranjo scale is unacceptable in suspected HILI cases, its results are heavily disputed (Liss and Lewis, 2009; Mahady et al., 2008; Sarma et al., 2008; Teschke, 2010c; Teschke and Wolff, 2011; Teschke et al., 2011d,e; Teschke and Schulze, 2012); this also pertains to a shortened version with only 5 of the original 10 items (Teschke and Schulze, 2012). Lack of hepatotoxicity specificity of the Naranjo algorithm was associated with missing definition of liver ADR, unclear time frame and latency period, undefined time frame for dechallenge, lacking risk factor definition, insufficient evaluation of alternative diagnoses, inappropriate assessment of comedication; and missing definition of a positive rechallenge test (Naranjo et al., 1981; Teschke and Schulze, 2012). This scale also was considered insensitive, allowing a possible causality even in the absence of essential data by virtue of the patient simply having taken the suspected agent (Liss and Lewis, 2009; Sarma et al., 2008). The Naranjo scale as modified by USP (Mahady et al., 2008) did not exclude alternative causes such as idiopathic autoimmune hepatitis, alcoholic or cardiac hepatopathy, other preexisting liver diseases, DILI, and drug-induced rhabdomyolysis (Sarma et al., 2008; Teschke, 2014; Teschke et al., 2011d,e). It therefore appears that the USP approach (Mahady et al., 2008) is an invalid tool for causality assessment in suspected HILI, leading to the conclusion that quality of causality assessment is more important than quantity of counted cases, not vice versa (Teschke et al., 2012g). Use of this method has raised concern about judgment validity by the USP regarding cases of hepatotoxicty by green tea (Liss and Lewis, 2009; Teschke and Schulze, 2012).

#### WHO method

The WHO method in short consists of both the WHO scale and the global introspection by experts (WHO, 2000b) and was applied for assessing causality in cases of kava hepatotoxicity by the WHO (2007a) and of PS hepatotoxicity as erroneously assumed by the German regulatory agency BfArM and the Drug Commission of the German Medical Association (DCGMA, 2011), but the value of this hepatotoxicity unspecific method was heavily debated (Stammschulte and Gundert-Remy, 2012; Teschke et al., 2012b,c,d) and judged obsolete before (Teschke and Wolff, 2011), considering its known shortcomings (Table 13). In general, global introspection represents a strategy in evaluating the likelihood of drug causality for adverse reactions (Kramer, 1986). Surprisingly, this method also has never been validated for any ADRs (Teschke et al., 2012c); as early as 1986, global introspection by experts has been shown to be neither reproducible nor valid (Kramer, 1986). Both the questions and the answers are ambiguous (Teschke and Wolff, 2011). Specifically, the assessor considers factors that might causally link one or more drugs to an observed ADR, lists all factors, weighs their importance, and decides the probability of drug causation (Kramer, 1986). No specific check list or level of strength is given.

The WHO scale was not validated by a gold standard, is not quantitative, not specific for hepatotoxicity (WHO, 2000b; Teschke and Wolff, 2011; Teschke et al., 2012b,c,d, 2013b,e). Reliability, sensitivity, specificity, positive and negative predictive values are unknown. Its scope is also limited since it cannot discriminate between a positive and a negative correlation, thereby stimulating overdiagnosing and overreporting (Teschke et al., 2013b). The WHO method ignores uncertainties in daily dose, temporal association, start, duration, and end of herbal use, time to onset of ADR, and course of liver values after herb discontinuation. Insufficiently considered or ignored are comedications, pre-existing liver diseases, numerous alternative explanations, and exclusion of virus infections by hepatitis A - C, CMV, EBV, HSV, and VZV (Teschke et al., 2012b,d). Similarly, case duplications and retracted cases remained undetected by the WHO method (Teschke et al., 2012a). Despite these flaws, the WHO method was used for causality assessment in herbal hepatotoxicity cases (Elinav et al., 2007; Schoepfer et al., 2007; DCGMA, 2011; Stammschulte and Gundert-Remy, 2012; Teschke et al., 2012b,d); claimed causality for PS was not confirmed after reevaluation in two studies (Teschke et al., 2012b,d).

#### Other approaches

Other attempts to evaluate causality in assumed HILI cases exist (Hung et al., 2011), also the ad-hoc assessment (Kaplowitz, 2001), which was preferentially used for kava cases by the German regulatory agency (BfArM, 2002) and in detail disputed subsequently (Teschke and Wolff, 2011), and the Karch & Lasagna method applied in some HILI cases of Herbalife® and considered obsolete recently (Teschke et al., 2013d), due to known shortcomings.

#### Questionable and lacking causality

Although causality was firmly established for various herbal TCM preparations as well as other herbs and herbal products in reported HILI cases (Tables 1, 2) (Teschke et al., 2011a, 2012a,e, 2014a), causality problems emerged with a few herbs and herbal preparations as evidenced by some full length published reports with detailed analyses. Among these are black cohosh with a possible causality grading in one single HILI case (Teschke, 2010c) and lacking causality in another study (Naser et al., 2011), kava with a highly probable causality level in one HILI case confirmed by a positive reexposure test result (Teschke et al., 2008a), and a confirmed causality grading assessed by a positive reexposure test result for a Herbalife® product in a single HILI case (Teschke et al., 2013d); however, CIOMS/RUCAM based causality for some Herbalife® products was highly probable in one patient and probable in six patients, as preliminarily reported in abstract form without any case details including case data quality (Halegoua-De Marzio et al., 2013), which was described as poor and scattered before (Teschke et al., 2013c).

With Ba Jiao Lian (*Dysosma pleianthum*), this TCM herb was not further considered as hepatotoxic (Teschke, 2014; Teschke et al., 2014c, 2015b), since not all diagnostic criteria were fulfilled for cases of hepatotoxicity by this herb (NIH, 2014c; Teschke, 2014). In detail, after herbal use at recommended doses, the patients manifested abnormal liver function tests associated with nausea, vomiting, diarrhea, abdominal pain, thrombocytopenia, leucopenia, sensory ataxia, altered consciousness and persistent peripheral tingling or numbness. However, the increase of the aminotransferases was marginal, with preference of AST rather than ALT. The AST increase could reflect isolated damage of the mitochondria around the hepatic central vein or muscular damage, because of the associated increase of creatine phosphokinase, findings not in support for a clinically relevant toxic liver disease (Teschke, 2014). Evidence against a hepatotoxic potential was also provided for Jing Tian San Qi (*Sedum aizoon*) as another herbal TCM (Teschke, 2014), based on the results of recent studies showing that in patients with HSOS, the hepatotoxic PAs in the herbal TCM Tu San Qi (*Gynura segetum*) were responsible rather than the misidentified *Sedum aizoon* lacking these alkaloids (Dai et al., 2006; Gao et al., 2006, 2012; Wu et al., 2008; Lin et al., 2011; Wang and Gao, 2014).

#### Pathogenetic aspects of HILI

Any HILI case report should describe details to ensure a pathogenetic case classification, using appropriate criteria that characterize two major forms of HILI (Figure 1). One of these is named idiosyncratic, the other one intrinsic (Zimmerman, 1999; Teschke et al., 2008a). The idiosyncratic form of injury is unpredictable and independent of the dose; its metabolic and immunologic subtypes require special attention in clinical practice (Figure 1). Conversely, the intrinsic form of liver injury is predictable and dose dependent (Figure 1). Although, valid data are lacking, it appears that most HILI cases are of the idiosyncratic rather than the intrinsic form.

#### Idiosyncratic form

As an example, clinical assessment characterized kava hepatotoxicity as an idiosyncratic liver injury linked to a metabolic aberration in unusually susceptible humans, providing an overall low incidence of kava hepatotoxicity in the normal population (Teschke et al., 2008a). This rarity of kava hepatotoxicity was also considered in the recent kava trial and evaluated as a positive risk/benefit constellation (Court, 2014), opposing previous regulatory assumptions to the contrary (BfArM, 2002). In accordance with other HILI cases of the idiosyncratic form of injury, human kava hepatotoxicity is not reproducible in experimental animals. Therefore, results of preclinical assessments with kava in experimental studies showing lack of liver toxicity are not transferrable to humans with another susceptibility setting and are not suitable to ensure safe use in humans. Since experimental reproducibility is missing, the lack of an experimental model prevents analytical evaluations directed to a proposed molecular mechanism of kava hepatotoxicity. Regarding human kava hepatotoxicity, characteristics of the metabolic subtype of the idiosyncratic form of injury prevail, based on the variable duration of exposure of 1week up to 12 months, associated with a weak dose dependency (Teschke et al., 2008a). Overall, most plants are fairly well tolerated by humans, whether used as normal food, herbal drugs, or HDS.

The pathophysiology of idiosyncratic HILI in humans is difficult to assess due to lack of experimental reproducibility and hence missing existence of an experimental animal model of HILI. There are abundant studies related to effects of herbs on animals or in vitro cell systems, but uncertainty exists whether these experimental results are transferable to human idiosyncratic HILI conditions. However, pathogenetic aspects are well assessable for HILI cases of the intrinsic form, due to available animal models with experimental hepatotoxicity and the possibility of transferring their results to human conditions.

#### Intrinsic form

Germander (*Teucrium chamaedrys*) hepatotoxicity is a typical liver injury of the intrinsic form, since it is dose dependent and reproducible in mice (Larrey and Faure, 2011). Due to its experimental reproducibility in animals, the molecular pathogenesis of Germander hepatotoxicity can easily be studied in experimental hepatotoxicity and transferred to human Germander hepatotoxicity. Germander components are neoclerodane diterpenoids that are oxidized by the cytochrome P450 3A isoform into reactive metabolites. These deplete hepatic stores of glutathione and cytoskeleton associated protein thiols, form plasma membrane blebs, and cause apoptosis contributing to liver cell necrosis (Larrey et al., 1992; Larrey and Faure, 2011).

PAs are other good examples for the intrinsic form of liver injury, which again is clearly dose dependent, thereby predictable, and hence preventable. For herbs containing PAs, each consumer of these herbs is at a dose dependent risk developing HSOS as a specific entity of liver disease (Smith and Desmond, 1993; Sperl et al., 1995; Stillman et al., 1977; Fu et al., 2004). PA containing plants are probably the most common poisonous plants affecting not only humans but also livestock and wildlife, with more than 6.000 plants containing PAs and about 3% of the world's flowering plants containing PAs (Fu et al., 2004). Some of these plants have caused toxic liver disease, recognized as epidemics and sometimes primarily assigned to viral hepatitis and not necessarily to toxic plants (Tandon et al., 1976a,b, 2008). Human embryotoxicity caused by PAs has been described in a newborn whose mother drank one cup of a tea containing PAs per day throughout pregnancy (Roulet et al., 1988; Fu et al., 2004). Some PA containing plants such as *Crotalaria species* (Bush tea, Rattlebox), *Ilex paraguarensis* (Mate tea), *Symphytum species* (Comfrey), *Senecio species* (Groundsel), *Heliotropium species*, and *Compositae species* (Indian herbs) that caused HILI are tabulated (Table 2). These herbs also injure cattle and house animals (Fu et al., 2004) and cause experimental hepatotoxicity in animals (Lin et al., 2011). PAs can be quantified in the serum of patients with HSOS (Lin et al., 2011; Larrey and Faure, 2011). The pathogenesis of PA hepatotoxicity has been elucidated in experimental studies, which showed the involvement of hepatic microsomal cytochrome P450 in the activation of PAs (Larrey and Faure, 2011).

Finally, herbal TCM products containing more than 19 g dose of *Radix bupleuri* may increase the hepatotoxicity risk (Lee et al., 2011); this dose dependency was confirmed in experimental animals and provided insights into some pathogenetic processes (Liu et al., 2014).

#### Juristical considerations

#### Black cohosh

Legal aspects of HILI case assessment rarely provide particular juridical and clinical challenges. Two court decisions merit attention, one relates to BC and the other one to kava. In 2005, a report was published representing a case of a 50 year old woman with fulminant liver failure and liver transplantation in assumed connection with the use of BC (Levitsky et al., 2005). A product liability action was filed by the patient after her recovery (Nebraska, 2006).

The decision of the judge answered the question whether in the specific case under discussion sufficient evidence establishes the herb as a generally or individually specific cause for the observed liver disease; for black cohosh, both aspects of causation were denied. General causation refers to the previously established hepatotoxicity by the same herb, but this was denied because of lack of convincing data. Specific causation refers to the case under discussion; this was also refuted on grounds of conflicting case data, poor case data quality, and numerous confounding variables (Nebraska, 2006). Our clinical diagnosis in this case was herpetic hepatitis and liver disease unrelated to BC or comedicated drugs, and CIOMS based assessment led to an excluded causality for both BC and comedicated drugs (Teschke and Schwarzenboeck, 2009): For this case of BC overdose, conclusions for an update of 22.12.2006 were provided (EMA, 2007): Worst case causality scoring would be possible, if comments of the expert would not be taken into account; because of the clinical experience of the expert and the requested obligatory causality in front of an American court, preference is given keeping the causality at a probable level (EMA, 2007). The conclusions of EMA are cloudy, difficult to reconcile, and ignore presented details, since the judge actually excluded both involved experts from expert testimony as to causation according to Daubert, rule 702 (Nebraska, 2006). As explained in detail, this rule requires that an expert be qualified to render a testimony on the subject, and that his testimony be reliable and relevant. None of the experts obviously met these and other required qualifications. Following the trial, USP reduced the causality for BC in this case from a probable to a possible level (Mahady et al., 2008). An erratum clarified the case conditions (Levitsky et al., 2008). Our clinical diagnosis in this case was herpetic hepatitis and liver disease unrelated to BC or comedicated drugs; CIOMS based assessment led to an excluded causality for both BC and comedicated drugs (Teschke and Schwarzenboeck, 2009). In retrospect, this court case calls for a thorough transparent documentation of HILI cases, associated with an unbiased expert opinion.

#### Kava

Kava was in the focus of another trial in connection with its marketing withdrawal by the German regulatory agency BfArM (Schmidt, 2014). Almost 12 years after the German regulatory agency BfArM issued an intermediate withdrawal of marketing authorization for products containing extracts of kava (*Piper methysticum*, Piperaceae) root and/or rhizoma (BfArM, 2002), the case has been reviewed by the German administrative court in Cologne (Court, 2014). According to the court's ruling on June 11, 2014, there was no justification for the ban of kava medicinal products issued by the German BfArM (Schmidt, 2014). The court ruled that based on available evidence, the benefit/risk ratio of kava medicinal products was confirmed as positive and must be considered as positive (Court, 2014), with credit given for previous reports (Schmidt, 2014) assessing causality in cases of assumed kava hepatotoxicity with the CIOMS scale (Teschke et al., 2008a, 2010; Teschke and Wolff, 2009; Teschke, 2010a). Credit was also given (Court, 2014) to the work of others (Sarris et al., 2013). Their double blind, randomized, placebo controlled trial was performed with a well defined noble kava drug that is on the market in Australia, showing both efficacy of kava in patients treated for their generalized anxiety disorders and lack of overt adverse reactions (Sarris et al., 2013), confirming kava efficacy based on a previous Cochrane study (Pittler and Ernst, 2003a). As a consequence of the court's ruling, German kava products have been formally restored to their market status on June 2002. As an update, BfArM appealed the court's ruling on June 30, 2014, but justification for the appeal has not yet been published (Schmidt, 2014). Clearly, the ruling is a major breakthrough, as it strengthens the legal certainty and predictability of regulatory decisions for herbal medicinal product manufacturers in general. It is also a call for the BfArM and other regulatory agencies to present transparent and appropriate clinical documentations of future HILI cases to be evaluated by clinically well trained regulatory assessors and external experts in the field, to be more self-critically, not to dismiss expert views to the contrary a priori, and providing rather than refuting original HILI case data in anonymous form to interested requesting scientists to assist in case evaluations.

### Essentials of herbal product quality

#### Herb authentication and product identification

Good quality of herbal drugs and other herbal products is prerequisite for safe human use (Table 16) (Teschke et al., 2013c). However, shortcomings of herbal products are well documented, both in herbal TCM and herbal modern medicine. Herbal authentication was an issue for BC (Sarma et al., 2008; Health Canada, 2010) and various TCM herbs (Haller et al., 2002; Teschke, 2014; Teschke et al., 2014b). There also was considerable debate whether kava products used by patients with kava hepatotoxicity might have been of poor quality including inappropriate herb authentication. This led to a comprehensive assessment and a proposal for a Kava Quality Standardization Code (Teschke and Lebot, 2011). Actually, several guidelines exist already for Good Agricultural Practices (GAP) and Good Manufacturing Practices (GMP), applicable to medicinal plants and herbal medicines to ensure their product quality (WHO, 2000a, 2003, 2006, 2007b). Despite these official precautionary recommendations for quality improvements, batch and product variability is not unusual (Lebot, 2006; Schmidt, 2007; Teschke and Lebot, 2011; Teschke et al., 2013c). Violation of GAP or GMP rules also will result in herbal products that lack efficacy, safety, or both.

**Table 16:** **Proposal for international harmonization: requirements for regulatory approved herbal drugs**.

**Specific international qualification required for regulatory approved herbal drugs**
Good Agricultural Practices
Good Manufacturing Practices
Definition of plant family, subfamily, species, subspecies, and variety
Definition of plant part
Definition of solvents and solubilizers
Lack of impurities, adulterants, and misidentifications
Minimum of batch and product variability
Lack of variety to variety variability
Brand name with details of ingredients, plant parts, batch number, and expiration date
Manufacturer with address
Regulatory specification of indication of herbal drug use
Daily dose with details of the application form
Maximum duration of herbal drug use
Efficacy of the herbal drug proven by valid RCTs
Description of adverse reactions and their frequency
Information of risk/benefit profile
Internationally approved unified regulatory surveillance
Regulatory harmonization of updated CIOMS scale use to assess causality in suspected HILI
Placebo controlled randomized double blind clinical trials
Risk/benefit profiles

Data are derived and adapted from a previous report (Teschke et al., 2013c).

When plants are considered for human use as ingredients of a herbal drug and dietary supplement, a clear definition and identification of plant family, subfamily, species, subspecies, and variety is mandatory, best done by a professional classical botanical description for any herb. Neglect may cause variation in plant family and species, contributing to the overall batch and product variability. Appropriate information should be provided by the manufacturers in the consumer's leaflet, thereby being available to the physician who suspects liver injury induced by a herbal product. The leaflet requires the name of the herbal product and the manufacturer's address who will provide additional information upon request. Therefore, all essential data of herb identification and the herbal product should be available before reporting HILI case details as spontaneous reports to the regulatory agencies or as case report publication. However, pitfalls are evident already at this stage of case evaluation.

In kava drug hepatotoxicity as an example, herb identification problems were evident. The manufacturers did not provide details on kava variety identification, so this specific information was missing in all spontaneous reports and case report publications (BfArM, 2002; Schmidt, 2007; WHO, 2007a; Teschke et al., 2008a; Teschke, 2010a,b, 2011). In the South Pacific region of origin, several hundred kava varieties exist—also called kava cultivars—and are grouped into noble, medicinal, and Two-Day varieties (Lebot, 2006; Schmidt, 2007; Teschke and Lebot, 2011; Teschke et al., 2011b). They differ in their kavalactone composition and their pleasant and unwanted, possibly toxic effects. In cases of suspected hepatotoxicity, it remained unclear which kava variety had to be incriminated. Interestingly, regulatory approval of kava drugs neither considered different kava varieties nor required respective labeling (BfArM, 2002). Thus, kava hepatotoxicity remains unexplained.

Other problems of herb or product identification have been described in detail in various cases of initially suspected herbal hepatotoxicity (EMA, 2007; WHO, 2007b; Mahady et al., 2008; Teschke et al., 2011a,b,d,e). Incomplete herb description complicates accurate association of herbs with liver injury and allows only general assumptions (Teschke, 2010c; Teschke et al., 2013e,f). Besides overall herb descriptions, the brand name of the herbal product has been provided in only a few case reports, and data for manufacturer, plant part, and extraction solvent normally was fragmentary (Teschke et al., 2011e, 2013c). For instance, the rate of undetermined herbal products was 10/16 cases (63%) among published case reports (Teschke et al., 2011e). This high rate questions the validity of any causality attribution. Additional problems arise from herbal mixtures, in which individual ingredients are not specified (Teschke, 2010c; Teschke et al., 2011d,e). Again, case reports have been published as HILI even if the patients were not sure whether they used a herbal product at all (Teschke et al., 2011e).

For GC, case reports assumed causality for hepatotoxicity in all 21 cases, but details on the GC product were fragmentary (Teschke et al., 2012e). Out of these 21 cases, seven patients used a GC monopreparation and four patients a GC polyherbal product, brand names and manufacturers were known in only nine patients. Fears of liability may contribute to the restriction of detailed product specifications by the authors. On the other hand, the regulatory agency did not hesitate to provide all relevant data of GC products from spontaneous cases of GC hepatotoxicity (Teschke et al., 2011a).

Therefore, unless complete data for herbal identification, ingredients, and name of the herbal product are provided in each HILI case, a valid causality assignment is not realistic. Identification problems are evident also in most HDS providing little specific information (Navarro et al., 2014; Robles-Diaz et al., 2015).

#### Plant part specification

Reports on HILI rarely provide details of the plant part used, ignoring specific toxic properties attributable to different parts of a plant. The regulatory recommendation for kava drugs was to use its peeled rhizome (Teschke and Lebot, 2011). In various assumed HILI cases by kava it remained unclear, whether also unpeeled rhizomes, peeled and unpeeled roots, and/or stem peelings were used, hampering evaluation of the causative agent of kava hepatotoxicity (WHO, 2007a; Teschke, 2011). For the U.S. FDA, peeled kava rhizomes were recommended for kava supplements (Teschke and Schulze, 2010), and according to the Australian Therapeutic Goods Administration, the commonly used medicinal kava products are derived from peeled rhizomes (Sarris et al., 2009). Plant part specification can be a major regulatory, agricultural, manufactural, pharmaceutical, and clinical issue (Teschke and Lebot, 2011).

#### Solvents and solubilizers

Herbal drugs and supplements are specified as extracts that are either water based or prepared from organic solvents like ethanol or acetone, but regulatory advice is often lacking (Teschke and Lebot, 2011). Thus, herbal extracts will substantially differ in their composition depending on the solvent. In addition, numerous solubilizers like macrogol, craspovidon, mentha oil, methyl acrylic acid polymer and polysorbate polyols may be included in herbal products to facilitate gastrointestinal uptake (Teschke, 2010b). Therefore, solvents and solubilizers may influence the composition of chemicals in the herbal product and selectively affect the bioavailability for the liver as the target organ. These variations hamper causality attribution in suspected HILI cases, leading to the recommendation that kava drugs and supplements should be water based extracts lacking any solvents or solubilizers (WHO, 2007a; Teschke and Schulze, 2010; Teschke et al., 2011b).

#### Misidentifications, impurities, and adulterants

For herbal product quality, not only plant misidentification but also contaminants, impurities and adulterants still remain key problems (Kang-Yum and Oransky, 1992; Espinoza et al., 1995; Gertner et al., 1995; Huang et al., 1997; Ko, 1998; Ernst, 2002; Estes et al., 2003; Lebot, 2006; Schmidt, 2007; Seeff, 2007; WHO, 2007b; Mahady et al., 2008; Navarro, 2009; Teschke et al., 2009, 2011c,e; Health Canada, 2010; Larrey and Faure, 2011; Teschke and Lebot, 2011). Adulterants are not uncommon in herbal TCM mixtures; they usually consist of synthetic drugs to provide or fortify product efficacy. Although rarely addressed by analytical approaches in patients with actually reported HILI, Health Canada was the only regulatory agency with recently reported interest in the analytical assessment of herbal products to evaluate quality, providing evidence for misidentification of herbs in some products and presenting results that the accused herb was not present in the herbal products used by the affected patients (Health Canada, 2010).

It remains to be established to what extent misidentifications, impurities, and adulterants are responsible for individual HILI cases. For instance, possible causality for hepatotoxicity cases of the herbal TCM mixtures Chaso and Onshido was ascribed to N-nitroso-fenfluramine, found as adulterant in these slimming aid products that had been produced in China and sold in Japan (Adachi et al., 2003). However, there is only little clinical or experimental evidence for a potential hepatotoxicity by this adulterant (Kanda et al., 2003a,b; Lau et al., 2004). It rather appears that green tea as ingredient was the causative agent if supplied as extract (Teschke, 2014).

Misidentification may create major clinical challenges and harm dramatically the health of consumers, shown for the following cases (Teschke, 2014). Until 2008, overall 41 cases from China with HSOS, the former HVOD, were reported and causally attributed to the herbal TCM Jing Tian San Qi (*Sedum aizoon*, syn. Stonecrop) (Wu et al., 2008), but causal attribution to *Sedum aizoon* was obviously incorrect. *Sedum aizoon* lacks PAs, and when applied to experimental animals, HSOS did not eme.g., Lin et al., 2011, suggesting that a herb containing PAs likely is reponsible for the reported cases (Wu et al., 2008). In line with this is another hepatotoxicity case from Hong Kong with HSOS that initially also was ascribed to *Sedum aizoon*, but it turned out to have been caused by the herbal TCM Tu San Qi (*Gynura segetum*) (Lin et al., 2011). The name and appearance of *Sedum aizoon* is similar to the one of *Gynura segetum*, but botanical differentiation was considered possible for the eye of experts (Lin et al., 2011). Comparative studies with both herbs provided clear supportive evidence for *Gynura segetum* as culprit for additional cases of HSOS as compared to *Sedum aizoon*. Respective studies in mice showed that *Gynura segetum* as the PA containing herb but not *Sedum aizoon* lacking PAs causes experimental HSOS as assessed by liver histology results (Lin et al., 2011). In an earlier experimental study, a model of the hepatic veno-occlusive disease was established by PAs derived from a herb described erronously as *Sedum aizoon* (Gao et al., 2006), which again does not contain PAs (Lin et al., 2011; Gao et al., 2012; Wang and Gao, 2014). This suggests that the described experimental model (Gao et al., 2006) was due to the action of a herb containing PAs, most likely *Gynura segetum* (Lin et al., 2011; Gao et al., 2012; Wang and Gao, 2014), rather than to *Sedum aizoon* lacking PAs (Gao et al., 2012). Based on these well founded considerations, evidence for a hepatotoxic potential of Jing Tian San Qi is lacking. The herbal TCM *Sedum aizoon* should therefore not be tabulated any more as hepatotoxic herb, as done until recently (Teschke et al., 2012h).

*Gynura segetum* was involved in other cases of herbal misidentification. In two Chinese women, HSOS emerged, which was induced by PAs of the herbal TCM *Gynura segetum* (syn. Ju Shan Qi, Ju Ye San Qi, Shan Chi, San Qi Cao, Shan Chi, Shan Chi) (Dai et al., 2006). Additional six cases were earlier suspected (Kumana et al., 1983, 1985); in at least four cases, the culprit was the PA containing herb *Heliotropium lasiocarpum* rather than *Gynura segetum* (Culvenor et al., 1986).

## New encouraging steps

Modern medicine is well established on our globe but provides health facilities only to parts of the population with focus on patients who can afford the expenditures. Challenges of modern medicine include management of chronic disorders and orphan diseases, all at reasonable costs. To achieve this goal, support may come from herbal medicine, but this will require major efforts at various levels. Since abundant plants grow in all countries around the world and are ready to be used for treating human diseases, herbal medicine may have encouraging perspectives to become a global player, provided efficacy is proven and associated risks such as liver toxicity are limited and easily recognizable.

### Progress in developing valid diagnostic biomarkers

Numerous valid clinical biomarkers exist and enable a firm diagnosis of most liver diseases unrelated to HILI and DILI, for instance by assessing specific antibodies of viral hepatitis (Table 7). New encouraging steps with the development of specific biomarkers for HILI are discussed (Larrey and Faure, 2011) in reference to a sensitive and specific assay enabling fthe detection of a reactive pyrrole-protein adduct in the serum of patients with HSOS. This disease was attributed to the Tusanqi preparation made erroneously with *Gynura segetum* containing PAs instead with *Segetum aizoon* lacking PAs (Lin et al., 2011). The results of this assay show that the patient actually consumed a herb containing PAs, which are metabolized in the liver to a reactive PA metabolite, reacting with a protein and forming an adduct (Larrey and Faure, 2011). However, this assay does not prove that PAs have caused the hepatotoxicity in this particular patient, needing supportive evidence in the clinical context. Measuring herbal toxins or their metabolites in the serum is useful in HILI cases in a setting of some intoxication, if high levels of the herbal toxin are expected in the serum due to large amounts of the consumed herb, high cumulative doses, or a prolonged degradation of the toxic herbal chemical. These conditions apply to HILI cases of the intrinsic form but not to those of the idiosyncratic form, which accounts for most HILI cases. For idiosyncratic HILI, similar restrictions apply regarding circulating micro-RNA (mRNA), presently investigated in intrinsic DILI and detectable in fluids including the serum (Zhou et al., 2013). Omics technologies, including genomics, proteomics, and metabolomics might well change but not revolutionize our understanding in the diagnosis of intrinsic hepatotoxicity (Yang et al., 2012b).

Interest in biomarkers to identify idiosyncratic hepatotoxicity risks in individuals who use drugs is continuing. For idiosyncratic DILI, numerous genetic and nongenetic risk factors have been described as possible biomarkers to predict DILI in some individuals (Chalasani and Björnsson, 2010), but whether these are useful to diagnose idiosyncratic HILI is unknown.

### Conformed diagnostic HILI case management

At an international level and to provide transparency and comparability, an overall accepted pragmatic stratification of HILI case assessment and data presentation should be adopted and more enforced. Sequential case management and presentation should focus on narrative case details for an overview of clinical features. This kind of information is best provided as table, easily also published even for a high number of cases, as illustrated for 16 HILI cases in a single report (Table 3); this facilitates HILI characterization caused by a single herb such as GC (Table 4). Detailed presentation of established criteria of hepatotoxicity definition, differentiation of hepatocellular, cholestatic, and mixed form of liver injury, and pathogenetic classification should be mandatory (Figure 1). Information of provided or missed case details and diagnostic parameters are valuable tools that signify case data quality and ensure transparency (Table 6). The concept of a sequential diagnostic approach in suspected HILI cases best starts with thorough clinical case assessments, subsequently combined with the use of the updated CIOMS scale (Tables 10, 11) as the mainstream tool (Tables 12, 13), and followed by expert opinion, if uncertainty remains. This worldwide applicable strategy allows transparency and provides a quick basis for final causality assignments of individual HILI cases by calculating individual and final scores of individual CIOMS items (Tables 10, 11, 15). This strategy of diagnostic harmonzation is pragmatic, time and cost saving, and facilitates potential reassessment by other clinicians, scientists, manufacturers, or regulatory agencies.

### International harmonization of regulatory efforts and surveillance

Encouraging efforts are reported from the Chinese State Food and Drug Administration (SFDA), progress is underway to improve regulatory surveillance of TCM herbal products (Zhang et al., 2012). In 2012, the SFDA model of safety monitoring and risk management of TCM drugs was still under exploration, with numerous regulatory and clinical issues. These include information on adulteration and counterfeit TCM drugs and clarification that except for SFDA approved Chinese and Western compound products, the addition of Western drugs into a TCM drug formula is illegal. SFDA has established examination methods and shelf sampling inspection of products in order to protect the safety of patients. It is not described whether SFDA proves causality in suspected HILI by TCM and CIOMS is used in analogy to other international registries and regulatory agencies (Tables 12, 14).

The regulatory situation of herbal medicines has thoroughly been evaluated worldwide for most countries of all continents (WHO, 2005). For herbal supplements, regulatory control varies among countries and commonly is less stringent or missing, whereas regulatory approved herbal drugs in Europe are under strict regulatory surveillance, as are approved synthetic drugs (EMA, 2014; MHRA, 2014). Regulatory efforts regarding herbal medicine products should be advanced, aiming at an identical quality level in all countries. This harmonization is best achieved by regulatory lifting all HDS to the level of herbal drugs, provided new regulations are formulated and strictly followed, and preclinical and clinical safety as well as efficacy is proven. Consumers will benefit from worldwide pharmacovigilance harmonization and quality control standards of herbal drugs (Table 16), deviced as previously outlined for kava quality standards (Teschke and Lebot, 2011).

### Sophisticated evidence based trials

China is the country with an extremely high number of published randomized controlled clinical trials (RCTs) (Wang et al., 2007), evaluating herbal TCM, but their efficacy has rarely been established due to poor study quality (Manheimer et al., 2009; Teschke et al., 2015a,b). There is increasing awareness that valid evidence based clinical trials for any herbal treatment should be mandatory as shown for kava through a Cochrane analysis (Pittler and Ernst, 2003a), associated with a robust risk management and balanced risk/benefit profiles (Tang et al., 1999; Wang et al., 2007; Manheimer et al., 2009; NIH, 2014a,b; Teschke et al., 2015a,b). For Europe, these trials are commonly required by EMA and national regulatory agencies for herbal drug approvement; this established system should be adopted by the WHO for global harmonization.

### Promising new drug research and development

Plants are natural producers of chemical substances, enforcing great expectations that in the future more synthetic drugs are developed based on herbal ingredients being effective in human diseases. In fact, for most of history, herbal medicine was the only available medicine. It has also been estimated that one third to one half of currently used drugs were originally derived from plants (Bent, 2008). Encouraging developments are underway (Pelkonen et al., 2014), with focus on herbal TCM (Li et al., 2012; Zhao et al., 2012).

### Globalization

With pragmatic modern drug medicine in competition, herbal medicine should contribute some of its items to a modern type of drug medicine as part of globalized health care systems at reasonable costs. On a long run, this approach appears feasible, provided traditional and modern herbal medicine conform to the expectations and needs of patients and consumers (Leonti and Casu, 2013), requiring new steps and improvements based on debated issues outlined above. Globalization of herbal medicine needs unrestricted exchange of herbal drugs among all countries. To ensure worldwide high quality, each herbal medicine should undergo strict regulatory surveillance and be classified as herbal drug according to internationally agreed criteria. Among these are strict adherence to GAP and GMP items, clear definitions of plants, plant parts, and solvents to be used, well defined indications and treatment modalities, proof of evidence based efficacy for the proposed indications, evaluation of adverse reactions, and positive risk/benefit profiles (Table 16). Presently, HDS fulfill these criteria only marginally and need a move to the herbal drug category; otherwise withdrawal from the market will be the alternative. Concern exists that presently limited scientific evidence exists to establish the safety and efficacy of most herbal products (Bent, 2008). Of the top 10 herbs in the United States, five herbs (ginkgo, garlic, St. John's wort, soy, and kava) have scientific evidence suggesting efficacy, but concerns over safety may temper the decision to use these products. Consequently, herbal products are not likely to become an important alternative to standard medical therapies or a global player, unless there are changes to the regulation and standardization of these products (Bent, 2008).

## Concluding remarks

Herbal use is common in traditional and modern medicine and requires more international harmonization to promote herbal medicine to a global player. In analogy to European regulatory settings, herbal medicinal products should be manufactured, marketed, and supervised as regulatory approved drugs similar to synthetic drugs. Certainly, this requires global consent and major efforts but it could open a global market for these drugs. International agreement should be reached on various issues, including best quality of herbal drugs, definition of indications, and proof of therapeutic efficacy by clinical trials, determination of therapy modalities such as posology, and assessment of adverse reactions in line with risk/benefit profiles. Present issues focus on poor herbal product quality, lack of proven efficacy, and rare adverse reactions including hepatotoxicity. These should be better recognized in the future by thorough clinical evaluation associated with the CIOMS scale as the best recognized causality assessing tool worldwide, possibly followed by expert opinion if uncertainty remains.

### Conflict of interest statement

The authors declare that the research was conducted in the absence of any commercial or financial relationships that could be construed as a potential conflict of interest.
